# Photothermal Nano-Vaccine Promoting Antigen Presentation and Dendritic Cells Infiltration for Enhanced Immunotherapy of Melanoma via Transdermal Microneedles Delivery

**DOI:** 10.34133/2022/9816272

**Published:** 2022-09-02

**Authors:** Jiaojiao Zhu, Ruimin Chang, Benliang Wei, Yao Fu, Xiang Chen, Hong Liu, Wenhu Zhou

**Affiliations:** ^1^Xiangya School of Pharmaceutical Sciences, Central South University, Changsha, Hunan 410013, China; ^2^Department of Dermatology, Hunan Engineering Research Center of Skin Health and Disease, Hunan Key Laboratory of Skin Cancer and Psoriasis, Xiangya Hospital, Central South University, Changsha, 410008 Hunan, China; ^3^National Engineering Research Center of Personalized Diagnostic and Therapeutic Technology, Xiangya Hospital, Central South University, Changsha, 410008 Hunan, China; ^4^Department of Thoracic Surgery, Xiangya Hospital, Central South University, Changsha, Hunan 410008, China

## Abstract

Immunotherapy has demonstrated the potential to cure melanoma, while the current response rate is still unsatisfactory in clinics. Extensive evidence indicates the correlation between the efficacy and pre-existing T-cell in tumors, whereas the baseline T-cell infiltration is lacking in low-response melanoma patients. Herein, we demonstrated the critical contribution of dendritic cells (DCs) on melanoma survival and baseline T-cell level, as well as the efficacy of immunotherapy. Capitalized on this fact, we developed a photothermal nano-vaccine to simultaneously promote tumor antigens presentation and DCs infiltration for enhanced immunotherapy. The nano-vaccine was composed of polyserotonin (PST) core and tannic acid (TA)/Mn^2+^ coordination-based metal-organic-framework (MOF) shell for *β*-catenin silencing DNAzyme loading, which was further integrated into dissolving microneedles to allow noninvasive and transdermal administration at melanoma skin. The nano-vaccine could rapidly penetrate skin upon microneedles insertion and exert a synergistically amplified photothermal effect to induce immunogenic cell death (ICD). The MOF shell then dissociated and released Mn^2+^ as a cofactor to self-activate DNAzyme for *β*-catenin suppression, which in turn caused a persistent CCL4 excretion to promote the infiltration of DCs into the tumor. Meanwhile, the liberated PST core could effectively capture and facilitate tumor antigens presentation to DCs. As a result, potent antitumor efficacies were achieved for both primary and distal tumors without any extra treatment, indicating the great promise of such a nano-vaccine for on-demand personalized immunotherapy of melanoma.

## 1. Introduction

Malignant melanoma is a type of lethal skin cancer with high morbidity and mortality rates, and the worldwide incidence has increased over the last few years. It originated from the transformation of melanocytes into malignant cells [1]. Conventional therapies for melanoma include surgery, chemotherapy, and radiotherapy, while the therapeutic benefits are unsatisfactory, usually resulting in treatment failure. Fortunately, the rapid advancement of immunotherapy provides a new hope for metastatic melanoma patients, which has revolutionized the field of melanoma therapy [2]. Currently, a few immune checkpoint inhibitors (ICIs), such as ipilimumab and nivolumab, have realized bench-to-bedside clinical translation, showing long-term and durable benefits in a proportion of patients. However, the overall response rate of melanoma patients for ICIs is not high (only ~30%) [3, 4], likely due to the intrinsic tumor immunosuppressive microenvironment (TIME) and poor tumor immunogenicity [5, 6]. Moreover, it is estimated that ~50% of patients show primary or acquired resistance to ICIs [7, 8], accompanied by severe adverse effects in a subset of patients [9]. In addition, the high drug cost brings a significant financial burden to patients, which becomes an uncertain factor that affects the treatment outcome. Therefore, the broad clinical application of ICIs is limited, which spurs the development of alternative immune-regulating strategies with higher efficacy and fewer side effects for more cost-effective melanoma therapy.

Recently, great attention has been paid on nanomedicine to facilitate tumor immunotherapy, and several nano-adjuvants have been reported that can regulate the TIME and improve the tumor immunogenicity for multimodal tumor therapy [10–12]. For example, several photothermal nanomaterials have been demonstrated to not only eradicate tumors via direct photothermal therapy (PTT) but also promote antitumor immunity via their in situ vaccine-like functions to synergize immunotherapy [13–15]. Upon laser irradiation, the photothermal effect causes immunogenic cell death (ICD) and releases tumor-associated antigens (TAAs). In ICD, the damage-associated molecular patterns (DAMPs) molecules, including calreticulin (CRT) and heat shock proteins (HSPs), are expressed on the tumor cell surface, and high mobility group box 1 (HMGB-1) and adenosine triphosphate (ATP) are released from tumor cells, both of which improve the tumor immunogenicity [16]. Meanwhile, the nanomaterials mediate the delivery of antigens released from dying tumor cells into dendritic cells (DCs), promoting the intratumoral infiltration of cytotoxic T lymphocytes (CTLs) for immune response [17, 18]. Notably, photothermal regulation is rather suitable for skin cancer as the limitation of light penetration is circumvented due to the superficial position of the cancer tissue, while the advantages of low invasiveness, precise temporal and spatial controllability, and minimal side effects are maintained [19, 20]. As such, several photothermal nano-vaccines have been developed, and several of them have integrated multiple immune regulation models to increase the sensitivity of immunotherapy [21–23].

**Figure 1:**
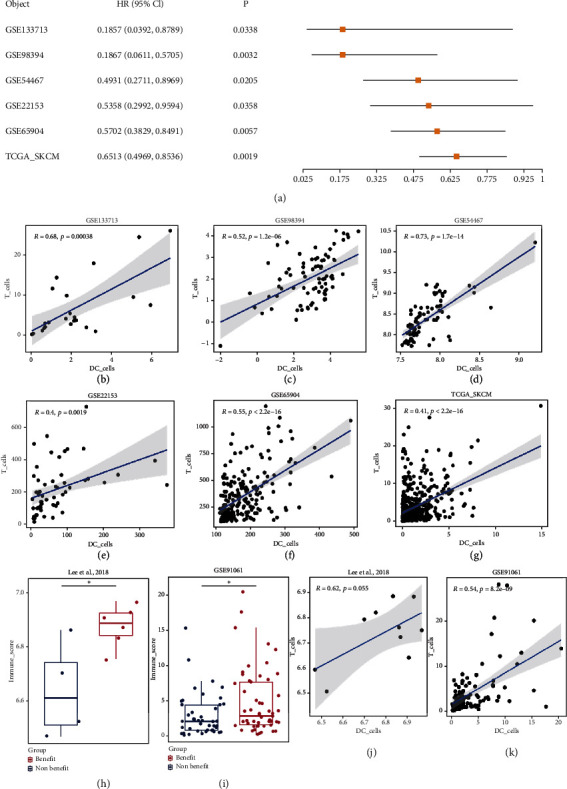
(a) In the melanoma cohort, the samples were grouped according to the median DC cells score and univariate COX regression analysis was performed. (b–g) Correlation analysis of DC cells and T cells in melanoma cohorts. There was a significant positive correlation between DC cells and T cells score. It suggested that DC cells recruited T cells to inhibit tumor. (h and i) Wilcoxon test was used to analyze DC cells score in the beneficial and non-beneficial groups, showing a significantly higher score in beneficial group. (j and k) Correlation analysis of DC cells and T cells in immunotherapy cohorts. There was a positive correlation between DC and T cell scores in the immunotherapy cohort.

During the process of cancer immunotherapy, DCs play indispensable roles as the most professional and potent antigen-presenting cells (APCs) in the body. DCs phagocytose, process, and present tumor antigens to T cells through the major histocompatibility complex (MHC), and the activated DCs could also regulate effector T cells recruitment via generating chemokine signals. Increasing evidence has indicated the correlation between intratumoral DCs (especially CD103^+^ DCs) and clinical outcomes [24–26]. Therefore, the level of DCs and the production of activated DCs are critical parameters to determine the efficacy of tumor immunotherapy. To figure out the functions of DCs in melanoma, herein we performed a systemic bioinformatics analysis, which revealed that DCs score was a protective factor of melanoma, and it was positively correlated with the baseline T cells level to determine the benefit of immunotherapy (Figure 1) [27]. However, the cunning tumor cells are adaptive to escape immune surveillance by inhibiting the infiltration and the activation of DCs, which severely restricts the effectiveness of immunotherapy [28]. To compensate the insufficiency of endogenous DCs, DCs-based immunotherapy has been attempted to implant exogenous DCs into the tumor. While this method is straightforward, the efficacy is sometimes limited due to the short survival time of the transplanted DCs as well as the low antigens presentation efficiency [29]. Therefore, strategies that can drive endogenous DCs recruitment in tumors are highly desired to turn cold tumor microenvironments into hot T-cell-rich areas. While DCs-targeting nanomedicines have been extensively explored, most of these studies focused on promoting antigen presentation and modulating DCs functions [30, 31]. Intratumoral delivery of chemokines (such as GM-CSF and CCL4) has also been explored to recruit DCs, but the delivery systems are complicated for keeping the activity and local release of chemokines, and transient recruitment DCs cannot effectively present tumor antigen [32–35]. Therefore, there still lacks of effective methods to achieve persistent DCs infiltration for long-last tumor immunotherapy.

Interestingly, it is reported that the Wnt/*β*-catenin signaling pathway is a melanoma-intrinsic oncogenic pathway that is involved in the recruitment of DCs [24]. Wnt/*β*-catenin pathway is an essential oncogenic signal concerning tumor development, progression, and immune evasion, which are upregulated in various cancer, especially melanoma and colorectal cancer [36, 37]. Dysregulation of the Wnt/*β*-catenin pathway reduces intratumoral D103^+^ DC numbers via suppression of CCL4 secretion in tumor cells (a chemokine that attracts DCs to infiltrate the tumor), thus presenting tumor-specific T cells priming [24]. Over the past few years, significant research interest has been attracted to screen Wnt/*β*-catenin signaling inhibitors for tumor management, and several effective inhibitors (such as obatoclax, carnosic acid, porcupine inhibitors, and tankyrase inhibitors) have been discovered [38–41]. However, many of these compounds did not achieve the expected efficacy on tumor inhibition but rather caused significant toxicity in both clinical trials and animal models due to the critical role of Wnt/*β*-catenin in stem and progenitor cells for their proliferation and differentiation [42]. While the direct antitumor application has not succeeded yet, several Wnt/*β*-catenin signaling inhibitors show the potential for enhanced immunotherapy [39, 40], likely due to their capability to remodel tumor immune microenvironment, especially promoting DCs infiltration by recovering the excretion of CCL4.

**Scheme 1:**
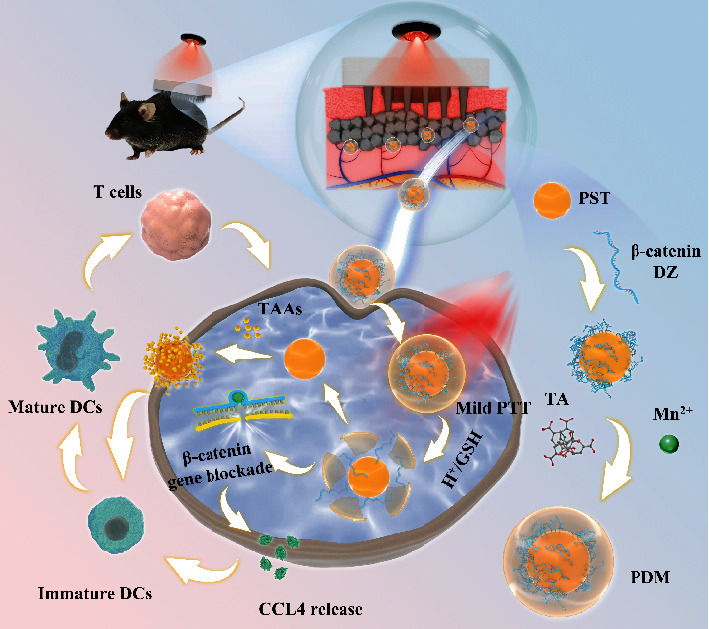
Schematic illustration of transdermal delivery of PDM via MNs patch for tumor immunotherapy. PDM caused tumor cells ICD upon laser irradiation, and the released TAAs were captured by PST to facilitate antigens presentation. Meanwhile, the self-activation of DZ silenced the *β*-catenin signaling pathway, which in turn promoted DCs infiltration via CCL4 excretion. Then, the matured DCs prime T cells leading to the activation of the adaptive immune response.

Given this fact, we designed a nano-vaccine to simultaneously promote tumor antigens presentation and DCs infiltration for boosting melanoma immunotherapy. The nano-vaccine (called PDM) was constructed by a polyserotonin (PST) core formed by autoxidation of serotonin monomer, with surface adsorption of *β*-catenin silencing DNAzyme (DZ) stabilized by a metal-organic-framework (MOF) shell via coordination between tannic acid (TA) and Mn^2+^ ion. PST is a mild photothermal agent, and we found that its photothermal conversion efficiency was markedly enhanced upon MOF shell layer coating. The DZ is a metal-dependent DNA catalysis, which could silence the target mRNA with high specificity and activity in the presence of its metal cofactor [43–47]. In our design (Scheme 1), the MOF shell provided a metal cofactor reservoir to release Mn^2+^ ions inside cells for the self-activation of DZ. To improve the efficacy and decrease the side effects [48], nano-vaccine was administered via transdermal microneedles (MNs) patch at the melanoma site and exerted a series of functions for enhanced immunotherapy: (i) PTT-induced tumor cells ICD to promote DAMPs and TAAs release; (ii) facilitating TAAs presentation via bio-adhesive nature of the PST core for antigens adsorption and intracellular delivery [49]; (iii) DZ-mediated Wnt/*β*-catenin silencing to improve DCs infiltration and maturation for better T cells activation. Such multi-functional nano-vaccine achieved a robust antitumor effect on both primary tumor and abscopal tumors without the need of extra therapeutic agents and showed excellent biocompatibility with no noticeable side effects during treatments.

## 2. Results and Discussions

### 2.1. Bioinformatics Analysis of the Influence of DCs on Melanoma

For rational nano-vaccine design, we first performed a systematic bioinformatics analysis to explore the correlation between DCs and melanoma. Univariate COX regression analysis showed that DCs score was significantly related to OS (Figure 1(a), Figure S1), and higher DCs score was a protective factor. Moreover, the DCs score was positively correlated with the T cells score (*R* >0, *P* < 0.05) (Figures 1(b)–1(g)), indicating critical roles of DCs for adaptive antitumor immunity. To confirm this, we further analyzed the correlation between DCs and the efficacy of immunotherapy, and an obvious positive association was observed (Figures 1(j) and 1(k)) [50–58]. In the immunotherapy cohort, the DCs score of the beneficial group was significantly higher than those of the non-beneficial group (Figures 1(h) and 1(i)). All these results demonstrated the importance of DCs infiltration for effective immunotherapy of melanoma and highlighted the potential benefits of DCs regulation to remodel tumor microenvironment. Capitalized on these facts, our nano-vaccine of PDM was designed with function to increase DCs infiltration in melanoma by regulating Wnt/*β*-catenin signaling pathway for enhanced immunotherapy.

### 2.2. Preparation and Characterization of PDM

**Figure 2:**
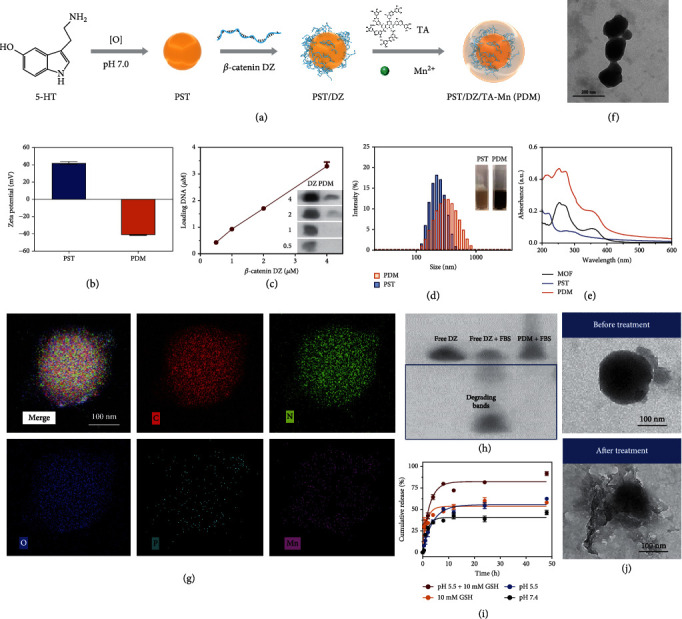
(a) Schematic illustration of the preparation of PDM. (b) The zeta potential of PST and PDM. (c) Gel electrophoresis quantification of DZ loading. Inset: gel images showing the band intensity of free DZ and PDM. (d) The size distribution of PST and PDM. Inset: corresponding photographs of PST and PDM solution. (e) UV-vis absorption spectra of PST, MOF, and PDM. (f) TEM image of PDM NPs. Scale bar =200 nm. (g) STEM-HAADF image and corresponding element mapping of PDM. Scale bar =100 nm. (h) PAGE gel image showing the DZ protection effect of PDM against the serum. (i) The release kinetics of DZ from PDM under various conditions. (j) TEM image of PDM before and after treatment with pH 5.5 PBS buffer containing GSH (10 mM). Scale bar =100 nm.

To prepare PDM, the PST core was first synthesized via oxidation and subsequent self-polymerization of 5-hydroxytryptamine (5-HT), and then the MOF shell was coated through the coordination between TA and Mn^2+^ (Figure 2(a)). PST exhibited positive surface charge due to the abundant amino group in its structure (Figure 2(b)), which allowed the adsorption of negatively charged DZ on the particle surface. The adsorbed DZ was further stabilized by TA/Mn^2+^ coordination. After DZ loading and MOF coating, the surface charge was reversed to negative. To quantify the DZ loading, DZ was labeled with FAM, which can be visualized on the gel image (Figure 2(c)). Upon encapsulation into PDM, however, DNA was retarded at gel loading well and the fluorescence was quenched by nanoparticles, and therefore, the DZ loading capacity can be calculated based on the intensity decrease. The nanoparticles could effectively encapsulate DZ with 80% loading efficiency at 4 *μ*M feeding DZ, attributable to the electrostatic attraction between PST and DZ, as well as the condensation effect of the MOF shell via metal coordination.

Upon MOFs coating, the PDM showed a noticeable color change and dynamic size increase (Figure 2(d)), and the characteristic UV-Vis absorbance (Figure 2(e)) of MOF was also seen. Such nanosystem showed high colloidal stability under various biological matrixes without remarkable particle change and aggregation over 3 days of incubation (Figure S2). From TEM, PDM showed a noticeable core-shell structure with particle size ~140 nm (Figure 2(f)). Elemental mapping was further performed to explore the composition, in which C, N, and O elements were seen throughout the structure while Mn element was only seen in the shell layer (Figure 2(g) and Figure S3). Notably, the P element was also observed in the peripheral region, confirming the DZ loading. As a type of nucleic acid-based drug, a critical challenge of DZ for biomedical application is in vivo degradation. We previously demonstrated that MOF could protect the loaded macromolecules (such as protein and nucleic acids) from enzymatic degradation [59], thus enhancing the biological stability of the payloads. To explore this, we treated the PDM with serum, and the DZ degradation was monitored by gel electrophoresis assay (Figure 2(h)). As a control, free DZ showed several degradation bands after 3 h incubation. After incubation with serum, PDM was treated with EDTA to release the DZ and then analyzed by PAGE gel. However, DZ remained intact with the single band in this case, verifying the protection effect of the MOF.

Besides stability, another issue of DZ for gene-silencing application is metal-dependent activity. The DZ used in this work was 8-17, which requires transition metal ions such as Mn^2+^ or Zn^2+^ as a metal cofactor [60]. To activate DZ, we employed Mn^2+^ as a metal ion for MOF formation and expected that such a structure could release Mn^2+^ after being delivered into cells for DZ activation. To demonstrate this, the release profile of Mn^2+^ and DZ was explored (Figure 2(i) and Figure S4). The release of Mn^2+^ can be triggered by both endo/lysosome mimic acidic environment and GSH, causing more than 50% Mn^2+^ release after 48 h incubation (Figure S4A). Similarly, the release of DZ was also accelerated by lowering the pH and adding GSH (Figure S4B). Such GSH- and pH-responsive release of both Mn^2+^ and DZ can be explained by the collapse of MOF structure under these conditions, which were evidenced by the morphology change after treatment (Figure 2(j)), as well as the significant decrease of surface charge (Figure S5). To further confirm Mn^2+^-mediated DZ activation, the released Mn^2+^ and DZ were collected and co-incubated with the mRNA substrate (Figure S6). A time-dependent cleavage was observed, in which ~70% substrate was cleaved in 8 h. Therefore, the MOF shell not only functioned for structure stabilization and DZ condensation but also served as a metal reservoir for DZ activation.

In addition to providing a surface for DZ adsorption, the other critical role of the PST core is to act as a photothermal agent. We previously have demonstrated the mild photothermal activity of both PST and TA/Mn^2+^-based MOF [59, 61]. Notably, PDM that combined PST and MOF showed a much stronger photothermal efficiency than each respective single structure (Figure S7A). In addition, PDM displayed a typical laser power- and concentration-dependent photothermal conversion capacity under 808 nm laser irradiation (Figure S7B, C), and also showed good photothermal stability and repeatability over five rounds of ON/OFF irradiation cycles (Figure S7D). To quantify the photothermal conversion efficiency (*η*), PDM was irradiated for heating and then naturally cooled to the original temperature, based on which the *η* was calculated to be 32% (Figure S7E). Collectively, the PDM could effectively load DZ with high colloidal stability, release Mn^2+^ and DZ in response to intracellular stimuli for DZ activation, and also display appropriate photothermal properties for tumor therapy.

### 2.3. Intracellular Functions of PDM: PTT-Mediated ICD, Tumor Antigen Presentation for DCs Activation, and *β*-Catenin Silencing to Promote CCL4 Excretion

**Figure 3:**
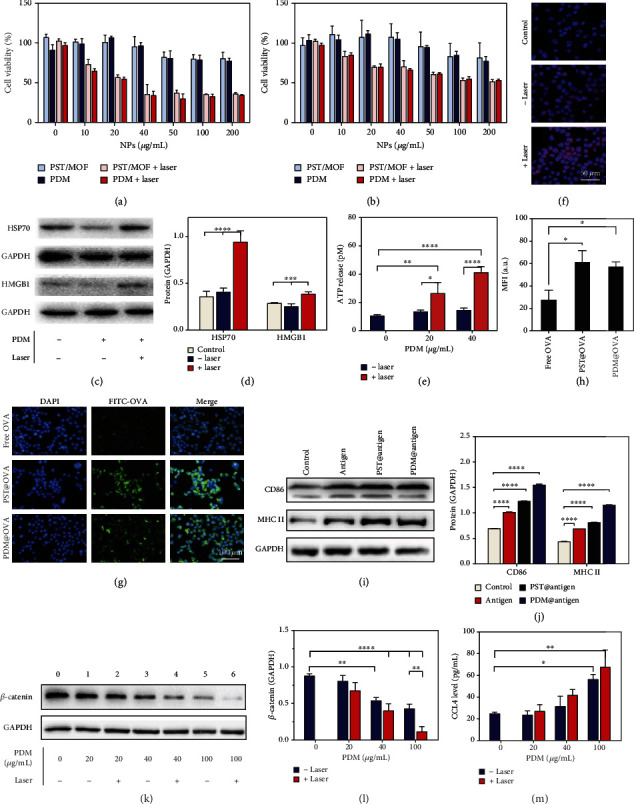
The viability of (a) B16F10 cells and (b) DC2.4 cells after various treatments. (c) Western blot analysis of the expression of HSP70 and HMGB1 of B16F10 cells after various treatments, and (d) the relative quantification of protein levels. (e) The level of ATP release after different treatments. (f) The fluorescence images show CRT expression. (g) Fluorescence images and (h) intensity quantification of OVA internalization. (i) Western blot analysis of the CD86 and MHC II expression in cells after different treatments, (j) and the relative quantification of protein levels. (k) Western blot analysis of the *β*-catenin expression in cells after different treatments, (l) and the relative quantification of protein level. (m) The CCL4 excretion after various treatments. The significance was defined as follows: ^∗^*P* < 0.05, ^∗∗^*P* < 0.01, ^∗∗∗^*P* < 0.001, ^∗∗∗∗^*P* < 0.0001.

After systematic characterization, we next moved to explore the intracellular functions of the nanoparticles. Based on the MTT assay on two cell lines of B16F10 tumor cells and DC2.4 dendritic cells (Figures 3(a) and 3(b)), both PDM and PST/MOF (the core-shell structure without DZ loading) were confirmed with high biocompatibility, in which >80% cells were viable at 200 *μ*g mL^−1^ nanoparticles after 24 h incubation. However, apparent cytotoxicity was observed upon laser irradiation attributable to the PTT effect, resulting in ~65% of tumor cells death at 40 *μ*g mL^−1^ nanoparticles. Notably, further increase of nanoparticle concentration failed to completely kill cells because of the limited efficacy of mild PTT. Interestingly, more B16F10 cells died under the same PTT condition than DC2.4 cells, indicating higher sensitivity of the tumor cells towards heating.

It is known that PTT-mediated cell damage could induce immunogenic cell death (ICD), which enhances the immunogenicity of tumors for better immunotherapy via the release of DAMPs and TAAs [18]. We then investigated the PDM-induced ICD by measuring various biomarkers, including the expression of HSP 70 and HMGB1 and CRT exposure, as well as ATP release. An obvious upregulation of both HSP70 and HMGB1 was observed at 20 *μ*g mL^−1^ TDM plus laser (Figures 3(c) and 3(d)), accompanied by an increase in ATP release (Figure 3(e)). The surface CRT level was visualized by the fluorescent staining, which was also intensified upon photothermal therapy (Figure 3(f)). PDM without laser, by contrast, did not cause much change for all these biomarkers, confirming the PTT-induced ICD. Collectively, PDM could damage a proportion of tumor cells, induce upregulation of CRT on the cell surface to provide an “eat-me” signal for antigen-presenting cells, and also express HMGB1/HSP70 as a secretory immunostimulant to prime the antitumor adaptive immune responses.

After tumor antigens exposure, the next critical step is effective antigen presentation for immune cells activation. By virtue of the bio-adhesive nature of both PST and MOF shells, we expected nanoparticles could adsorb the tumor antigens and facilitate their presentation [49]. To demonstrate this, we used the ovalbumin (OVA) as an antigen model to study its interaction with nanoparticles. After incubation with OVA, the surface charges of both PDM and PST were changed (Figure S8A), indicating surface adsorption of OVA. We then quantified the adsorbed protein by fluorescence intensity assay (Figure S8B). Specifically, OVA could be effectively adsorbed on the PST surface with a concentration of up to 1 mg mL^−1^. While the efficiency was much lower for PDM, this can be significantly enhanced after pretreatment (10 mM GSH, pH 5.5) to exfoliate the MOF shell layer (Figure 2(j)). Therefore, PDM could convert into PST in response to intracellular stimuli, which increases the capability to capture the released tumor antigens.

After demonstrating the tumor antigens capturing, we explored their capability for antigen presentation. To study this, OVA was labeled with a FITC fluorophore, which allowed direct observation by fluorescent microscopy. Free OVA showed only weak fluorescence in DCs, while the fluorescence was much brighter delivered by nanoparticles (Figure 3(g)). We also quantified the fluorescence, and a significant increase of intensity was observed for nanoparticles groups (Figure 3(h)), confirming the benefit of nanoparticles-mediated antigens delivery. We further investigated the activation and maturation of DCs after antigen presentation by measuring the expression of co-stimulatory molecules of CD86 and the antigen-presenting molecule of MHC II (Figures 3(i) and 3(j)). Compared with the free antigen, the nanoparticles@antigen complexes showed much better efficacy to promote the expression of CD86 and MHC II. Interestingly, PDM was more effective than PST in this case, likely due to the loose adsorption of antigen on the PDM surface that can be easily released from the surface for DCs activation. Overall, the nanoparticles could not only induce ICD for tumor antigens release but also facilitate antigens presentation for DCs activation.

In addition to antigens production and presentation, another critical function of PDM was to promote the excretion of CCL4 via silencing the Wnt/*β*-catenin signaling pathway. CCL4 is a type of chemokine that can recruit DCs into the tumor for adaptive tumor immunotherapy. However, its excretion is inhibited in melanoma cells due to the upregulation of Wnt/*β*-catenin, which develops an immune-suppressive tumor microenvironment. To reverse such effect, we employed DZ as a gene-silencing tool to knock down the *β*-catenin expression. Upon treatment with PDM, a concentration-dependent suppression of *β*-catenin was observed (Figures 3(k) and 3(l)). Interestingly, the DZ efficacy could be enhanced upon laser irradiation, which can be ascribed to the heating-induced enzymatic acceleration (Figure S9). CCL4 chemokine is the critical downstream signaling molecule, which is negatively regulated by Wnt/*β*-catenin. Upon *β*-catenin silencing by PDM, the excretion of CCL4 was promoted (Figure 3(m)). Therefore, PDM could release DZ inside cells for self-activation by Mn^2+^ and effectively down-regulate Wnt/*β*-catenin to promote CCL4 secretion, which in turn enhances the infiltration of immune cells for better immunotherapy.

### 2.4. Preparation and Characterization of PDM MNs for Transdermal Delivery

**Figure 4:**
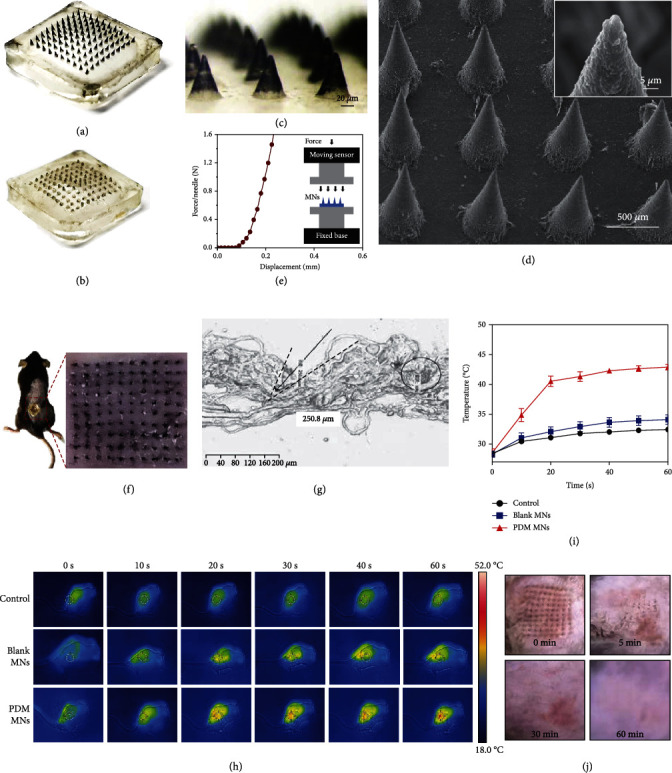
Digital images of (a) PDM MNs and (b) PDM MNs after inserting. (c) Micrograph of PDM MNs. Scale bar =200 *μ*m. (d) SEM image of PDM MNs in drying state. Inset: local view of MN tip. (e) Mechanical property of the PDM MNs. (f) Photo of mouse dorsal skin (the area within the red line) after being treated with one MN patch. (g) Image of frozen tissue sections to observe the cross-sectional area of mouse skin. The arrow indicates the insertion site. (h) Dynamic monitoring of the surface temperature changes of the MNs treated animal skin upon laser irradiation by a thermal infrared camera, and (i) the kinetics of temperature increase. (j) The appearance of the skin before and after MNs treatment at different time points.

To allow convenient administration, we next incorporated the PDM nanosystem into microneedles (MNs) for melanoma-targeted transdermal delivery. The MNs were prepared by using a commercially available micro-molding, in which PDM and the matrixes of hyaluronic acid (HA) and polyvinylpyrrolidone (PVP) were co-loaded into MNs with homogeneous size and morphology (Figure 4(a)). From both micrograph (Figure 4(c)) and SEM image (Figure 4(d)), a uniformed array of 10 × 10 MNs was assembled on a 2 × 2 cm^2^ patch, in which each MN had a conical construction at the base, a height of ~550 *μ*m, and a sharp tip tapering to a 5 *μ*m radius of curvature. To quantify PDM loading, the MNs were dissolved, and PDM was collected to determine the DZ amount, based on which each MN was calculated to contain 750 *μ*g PDM (~20 *μ*g DZ).

We then performed a systematic characterization of the MNs. The mechanical compression test was performed by measuring the force acting on MNs with an increase in MNs displacement (Figure 4(e)). The force reached ~0.8 N/needle when the displacement was 0.2 mm, suggesting a sufficient mechanical strength for skin insertion without needle breaking. We further applied the MNs to the abdominal skins of C57BL/6 mice (Figure 4(f)). After 2 min treatment, the skin insertion ratio reached 100%, and the insertion depth was 200~250 *μ*m according to histological examination (Figure 4(g)), which was deep enough to penetrate the superficial dermis of the skin. Such MNs could be rapidly dissolved 2 min after insertion (Figures 4(b) and 4(f)), which remained black dots (the color of nanoparticles) on the skin. The successful skin penetration of nanoparticles was further evaluated by in vitro transdermal experiments and in vivo thermal images. To analyze the penetration efficiency, a Franz diffusion cell system was employed. A time-dependent penetration of the nanoparticles was observed, in which ~70% PDM could penetrate the skin in 1 h (Figure S10). Therefore, the MNs could easily pierce the stratum corneum and dissolve quickly to release PDM for the transdermal drug delivery. At 24 h post-injection, the NIR laser (808 nm, 1.5 W cm^−2^) was applied to mice skin, and the temperature increase was recorded using a thermal infrared camera (Figure 4(h)). Compared with the control and blank MNs (the MNs without PDM loading), a significantly stronger temperature elevation was observed for PDM MNs within 1 min (Figure 4(i)), and such increment can be exploited for mild hyperthermia (42°C) of the tumor without side effects. In addition, the biocompatibility of MNs was evaluated by dynamically monitoring the skin after treatment, in which the majority of press traces faded and microchannels created by MNs were resealed at 30 min post-insertion, and the skin almost recovered to the original condition after 60 min (Figure 4(j)). Therefore, the skin could be quickly recovered after MNs treatment to minimize the entry of pathogens and infection risk. Moreover, the skins were further analyzed by H&E staining after various treatments (Figure S11), and no significant pathological change was observed after various treatments.

### 2.5. Antitumor Efficacy and Biocompatibility of PDM MNs In Vivo

**Figure 5:**
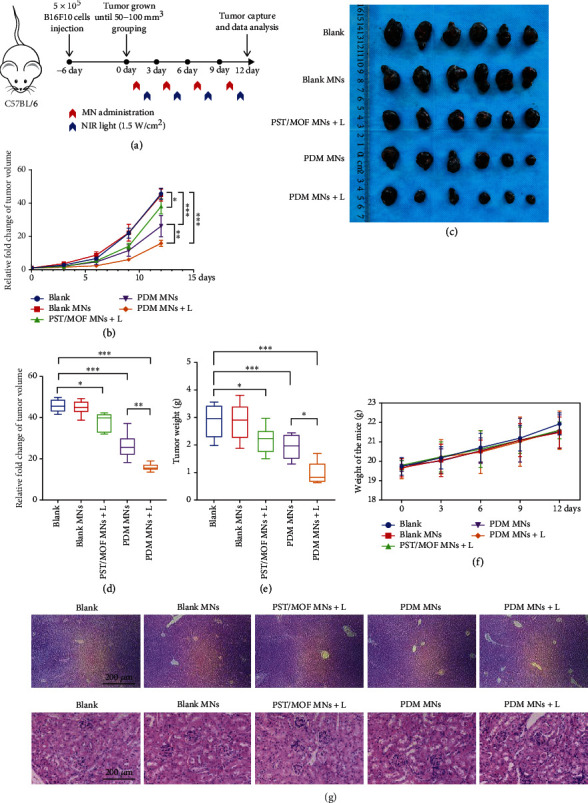
(a) Schematic depiction of the experimental approach for the antitumor treatment. (b) Tumor-growth curves of different groups. (c) Photos of tumors harvested from mice in each group on day 12. Relative (d) tumor volume and (e) tumor weight after various treatments. (f) The bodyweight of the mice during various treatments. (g) H&E staining images of liver and kidney after various treatments. The significance was defined as follows: ^∗^*P* < 0.05, ^∗∗^*P* < 0.01, ^∗∗∗^*P* < 0.001, ^∗∗∗∗^*P* < 0.0001.

We then studied the in vivo antitumor effect of PDM MNs by using a C57BL/6 melanoma model (Figure 5(a)). The mice with a tumor volume of 50~100 mm^3^ were randomly divided into 5 groups, each receiving one of the following treatments: blank MNs, PST/MOF MNs + laser (L), PDM MNs, and PDM MNs + L, respectively. The MNs patch was administrated on the dorsal skin region of the tumor, and the laser was performed at 24 h after MNs administration to allow sufficient skin recovery, tumor penetration, and intracellular delivery of the nanoparticles. The tumor volume was monitored every three days to dynamically evaluate the therapeutic outcome (Figure 5(b)). The blank MNs did not affect tumor growth, while a moderate antitumor effect was observed for PDM MNs, indicating the tumor inhibition efficacy of DZ via *β*-catenin downregulation. Upon laser irradiation, the PST/MOF loading MNs also triggered a marginal tumor suppression effect, which can be attributable to the mild PTT. Notably, the best efficacy was obtained for PDM MNs plus laser, suggesting a synergistic effect between PTT and DZ-mediated *β*-catenin silencing. After treatment, the mice were sacrificed, the tumor tissues were collected for direct observation, and the volume and weight were recorded for comparison, in which the antitumor activity was obtained in the order of PDM MNs + L > PDM MNs > PST/MOF MNs + L > blank MNs (Figures 5(c)–5(e)). Meanwhile, the biosafety of each treatment was evaluated. The body weight of all mice gradually increased, reflecting their good body condition during treatment (Figure 5(f)). After treatment, the major organs (including the liver and kidney) were harvested for H&E staining, and no significant physiological abnormality was seen (Figure 5(g)), confirming the high biocompatibility of the MNs for in vivo application benefiting from topical delivery.

### 2.6. Antitumor Mechanism of PDM MNs

**Figure 6:**
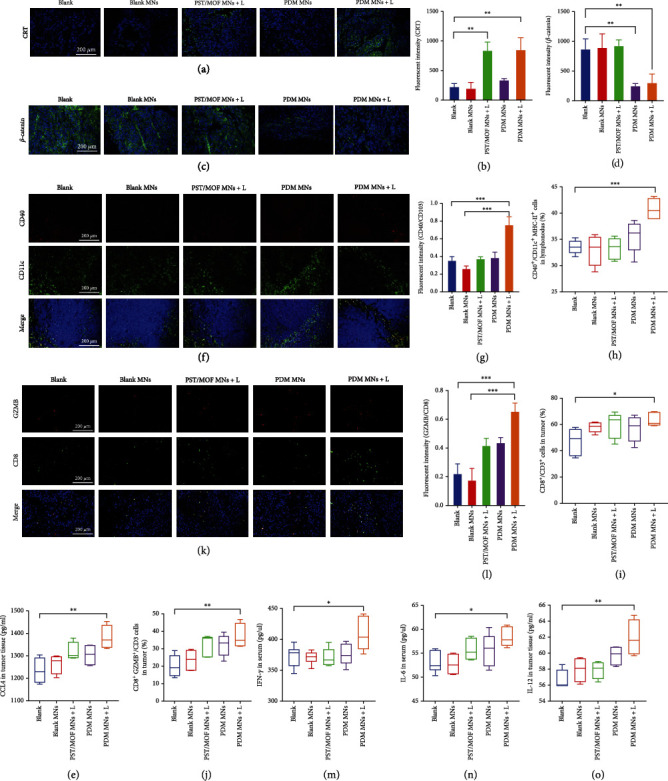
(a) Immunofluorescence staining of CRT (green) in tumor tissue and (b) the relative quantified results. Scale bar =100 *μ*m. (c) Immunofluorescence staining of *β*-catenin (green) in tumor tissue and (d) the relative quantified results. Scale bar =100 *μ*m. (e) The CCL4 level in tumor tissue. (f) Immunofluorescence staining of DCs (CD40^+^CD11c^+^) in tumor tissue and (g) the relative quantified results. Scale bar: 100 *μ*m. (h) The percentage of the matured DCs (CD40^+^CD11c^+^MHC II^+^) in lymph nodes. (i) The percentage of the CD8^+^ T cells (CD3^+^ CD8^+^) in tumor tissue. (j) The percentage of the GZMB^+^ CTLs (GZMB^+^ CD8^+^) in tumor tissue. (k) Immunofluorescence staining of GZMB^+^ CTLs (GZMB^+^ CD8^+^) in tumor tissue and (l) the relative quantified results. Scale bar: 100 *μ*m. (m) IFN-*γ*, (n) IL-6, and (o) IL-12 levels in sera and tumor tissue of mice. The significance was defined as follows: ^∗^*P* < 0.05, ^∗∗^*P* < 0.01, ^∗∗∗^*P* < 0.001, ^∗∗∗∗^*P* < 0.0001.

Having confirmed the robust antitumor effect, we next aimed to explore the underlying mechanism. In cellular studies, we demonstrated a systematic immune regulation effect of nanoparticles, which was achieved via promoting antigen release and presentation, as well as DCs recruitment via DZ-mediated *β*-catenin silence. We then studied all these effects in vivo. To probe tumor antigens presentation, the expression of CRT was measured by fluorescent staining. The CRT fluorescence markedly increased for both PST/MOF MNs and PDM MNs upon laser irradiation, demonstrating PTT-induced tumor cells ICD (Figures 6(a) and 6(b), Figure S12). The released tumor antigens could be captured by nanoparticles and then delivered into DCs for antigen presentation. Then, in vivo activation of DZ was evaluated by measuring the expression of *β*-catenin via both immunofluorescent and immunohistochemical assays (Figures 6(c) and 6(d), Figure S13). For the control group, the tumor tissue was characterized by a high *β*-catenin level. While both blank MNs and PST/MOF MNs had minimal effect on *β*-catenin expression, PDM MNs could strongly down-regulate *β*-catenin, thus increasing CCL4 levels in tumors (Figure 6(e)). Interestingly, the PST/MOF MNs could also promote CCL4 excretion to some extent, likely due to the positive effect of PTT-mediated ICD on antitumor immunity. As a result, the PDM plus laser produced the highest level of CCL4 by combining the efficacy of DZ activity and the PTT effect. It is known that CCL4 is the most crucial chemokine to recruit antigen-presenting cells into the tumor, mainly CD103^+^ DCs, which play a crucial role in recruiting T cells [24]. Thus, we further accessed the composition of immune cells in the tumors and lymph nodes, including CD103^+^ DCs, total CD11c^+^ DCs, total matured DCs, and various kinds of T cells. Recruited DCs usually specialize in acquiring tumor antigens in tumors and then presenting them to T cells in lymph nodes, which promote the infiltration and activation of T cells. Firstly, we access the level of matured CD103^+^ DCs (CD103^+^ CD11c^+^MHCII^+^) in lymph nodes by flow cytometry, and consistency with the release of CCL4, apparent increases were also observed in PST/MOF and PDM plus laser group. However, there was no significant increase in PDM group (Figure S14), which might be attributed to the low immunogenicity of tumors. We then measured the infiltration of total DCs (CD40^+^CD11c^+^) by immunofluorescence co-staining and flow cytometry, and as expected, the most abundant DCs infiltration was achieved for PDM plus laser group (Figures 6(f) and 6(g)). By virtue of antigen presentation and promotion of DCs infiltration, PDM MN plus laser significantly enhanced the CD40^+^ DCs level, which can activate CD8^+^ T cell. Such a process was probed by measuring the level of DCs maturation (CD40^+^CD11c^+^MHCII^+^) (Figure 6(h), Figure S15). Again, the PDM MNs plus laser group achieved the most significant DCs maturation among various therapies. Inspired by these results, we concluded that mild PTT and *β*-catenin DZ could synergistically enhance the infiltration and maturation of DCs.

As the final effector of immune cells, the infiltration and activation of T cells could reflect the efficacy of immunotherapy. The percentage of cytotoxic T lymphocytes (CTLs, CD3^+^CD8^+^) was analyzed by flow cytometry (Figure 6(i), Figure S16). Significant infiltration of CTLs was observed for both PST/MOF MNs plus laser and PDM MNs, owing to their respective regulation effect on tumor immunity, i.e., enhanced antigen presentation or immune cells infiltration. These two effects were combined for PDM MNs plus laser group, leading to an even higher level of CTLs. Upon activation, the CTLs could directly kill tumor cells by releasing various cytotoxins such as granzymes. We further analyzed the T cell activation by measuring the percent of granzyme B positive CTLs (CD3^+^CD8^+^GZMB^+^) by flow cytometry and immunofluorescence assay (Figures 6(j)–6(l), Figure S17). Consistent with the above observation, the PDM MNs plus laser group displayed the best ability to activate T cells. Moreover, several critical immunocompetent cytokines, such as IFN-*γ*, IL-6, and IL-12, were measured (Figures 6(m)–6(o)). These cytokines could not only directly damage tumor cells but also activate antitumor immunity for enhanced efficacy, all of which increased after PDM MNs plus laser treatment. Overall, both in vitro and in vivo evidence collectively substantiated that the PDM acted as a photothermal nano-vaccine, which was delivered by transdermal microneedles to exert antitumor effect via several cascade immune-regulation steps: promoting tumor antigen release by PTT-mediated tumor cells ICD; recruiting immune cells by DZ-mediated *β*-catenin silence to increase CCL4 release; facilitating antigen presentation by antigen capturing and engulfing. As a result, significant infiltration and activation of CTLs have been achieved to damage the tumor.

### 2.7. Abscopal Antitumor Effect of PDM MNs

**Figure 7:**
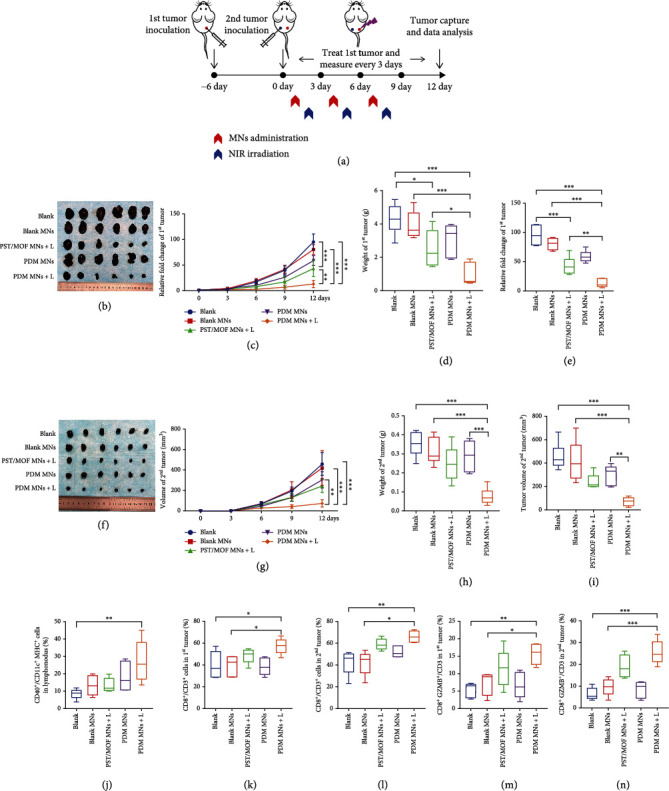
(a) Schematic depiction of the experimental approach for the abscopal effect of the PDM MNs. (b) Photos of primary tumors on day 12. (c) Tumor-growth curves of primary tumors, (d) the relative tumor weight, and (e) the relative fold change of tumor volume. (f) Photos of distal tumors on day 12. (g) Tumor-growth curves of distal tumors, (h) the relative tumor weight, and (i) the relative fold change of tumor volume. (j) The percentage of the matured DCs (CD40^+^CD11c^+^MHC II^+^) in lymph nodes. (k) The percentage of the CD8^+^ T cells (CD3^+^ CD8^+^) in primary tumor tissue. (l) The percentage of the GZMB^+^ CTLs (GZMB^+^ CD8^+^) in primary tumor tissue. (m) The percentage of the CD8^+^ T cells (CD3^+^ CD8^+^) in distal tumor tissue. (n) The percentage of the GZMB^+^ CTLs (GZMB^+^ CD8^+^) in distal tumor tissue. The significance was defined as follows: ^∗^*P* < 0.05, ^∗∗^*P* < 0.01, ^∗∗∗^*P* < 0.001.

Encouraged by the therapeutic efficacy of PDM MNs in the primary tumor, we are intrigued whether such systemic antitumor immune activation could be employed to control metastatic tumor, the main cause of death for most melanoma patients. To test this, a bilateral melanoma model was established, while treatments were only applied to the right side of the tumor (Figure 7(a)). The distal tumor was implanted at 6 days after the primary tumor. By following the same methods as described above, the tumor growth was dynamically monitored during treatments, and the tumor tissue was extracted after treatment for direct observation. Strikingly, while it is reasonable to see the growth inhibition for right-side tumors upon different therapies, the untreated left-side tumors were also suppressed to a different extent, indicating an abscopal effect. Note that the tumor suppression efficacy for both sides of tumors was quite comparable after various treatments, with the overall order of PDM MNs + L > PST/MOF MNs + L > PDM MNs > blank MNs (Figures 7(b)–7(i)). Therefore, the nano-vaccine could simultaneously manage primary tumor and distal tumor, which is beneficial for inhibiting tumor metastasis.

It is commonly accepted that the abscopal antitumor effect is mainly due to the activation of systemic antitumor immunity. To confirm such an effect, we first collected tumor-draining lymph nodes, and the percentage of matured DCs (CD40^+^CD11c^+^MHCII^+^) was measured (Figure 7(j), Figure S18). Compared with the control, the level of activated DCs markedly increased from 9% to 26% for PDM MNs plus laser, and both PST/MOF MNs plus laser and PDM MNs also triggered DCs activation, but to a lesser extent. Finally, the effector T cells were analyzed. For both primary and secondary tumors, the infiltration of CD8^+^ T cells significantly increased to 60% for the PDM-MN + L group, accompanied by the increase of the activated CTL (CD3^+^CD8^+^GZMB^+^) (Figures 7(k)–7(n), Figure S19). These results demonstrated that PDM-MN plus laser could induce a robust abscopal effect by promoting the infiltration and activation of T cells into the distant tumor.

## 3. Conclusions

In summary, we demonstrated the correlation between DCs and melanoma survival, T-cell baseline level, and response of immunotherapy, based on which a multi-functional yet straightforward nano-vaccine was constructed for enhanced melanoma immunotherapy via simultaneously promoting TAAs presentation and DCs infiltration. The nano-vaccine was facilely prepared and systematically characterized, displaying a protective effect for the loading DZ with a responsive release profile, as well as intrinsic photothermal activity. At the cellular level, the nano-vaccine could cause tumor cells ICD, capture the release TAAs for better internalization into DC cells, and realize the self-activation of DZ for *β*-catenin suppression to promote excretion of CCL4. For in vivo administration, the nano-vaccine was incorporated into MNs patch, which allowed conveniently transdermal delivery with melanoma targetability and low toxicity. Our experiments showed that this NIR-triggered nanosystem could elicit robust tumor-specific immunity to significantly suppress both primary and distant tumors, providing a therapeutic strategy to boost tumor immunotherapy with potential clinical applications.

## 4. Materials and Methods

### 4.1. Materials, Cells, and Animals

#### 4.1.1. Materials

Tannic acid (TA), 5-hydroxytryptamine hydrochloride (5-HT·HCl), hyaluronic acid (HA, MW =150-250 kDa), and polyvinyl pyrrolidone (PVP, MW =100 kDa) were purchased from Sigma-Aldrich (St Louis, MO, USA). Manganese chloride (MnCl_2_) was from Sinopharm Chemical Reagent Co., Ltd. (Shanghai, China). All DNAs were purchased from BioeGene (Shanghai, China). The DZ sequence was 5′-AAGGCGCATGATTTGTCCGAGCCGGTCGAAGGGCAAAGGGCAAG-3′, and FAM-labeled substrate strand (5′-CTTGCCCTTTGCCCrAGCAAATCATGCGCCTT-3′, rA indicates the ribonucleotide as the cleavage site). The ELISA kits were from Meimian Biotech (Wuhan, China). FITC-labeled OVA was from Beijing Solarbio (Beijing, China). ATP enhanced assay kit was from the Beyotime Institute of Biotechnology (Shanghai, China). Lipopolysaccharide (LPS, from *Escherichia coli* 0111:B4) was from Biosharp (Beijing, China). PE/Cyanine7 anti-mouse CD8a (100722), PerCP/Cyanine5.5 anti-mouse CD4 (100434), APC anti-mouse CD3 (100236), APC/Cyanine7 anti-mouse CD45 (103116), Zombie Aqua™ Fixable Viability Kit (423102), PE/Dazzle™ 594 anti-human/mouse Granzyme B Recombinant (372216), PerCP/Cyanine5.5 anti-mouse I-A/I-E (107626), APC anti-mouse CD11c (117310), PE/Cyanine7 anti-mouse/human CD40(124622), and Trustain fcX anti-mouse CD16/32 (101320) were purchased from Biolegend. Human CD8 (ab93278) and GZMB (ab134933) were purchased from Abcam. *β*-catenin (BS9828M) and CRT were purchased from Proteintech (27298-1-AP). CD40 (AF01782) and CD11c (AF07366) were purchased from AiFang biological (Changsha, China). All cellular experiment used reagents were purchased from Gibco Life Technologies (Grand Island, NY, USA).

#### 4.1.2. Cells

B16 melanoma F10 cells (B16F10) and DC2.4 cells were obtained from the Xiangya cell center (Changsha, China) and were cultured in contained 10% fetal bovine serum (FBS) and 1% penicillin (50 U mL^−1^) and streptomycin (50 U mL^−1^) Dulbecco's modified Eagle's medium (DMEM) in a 5% CO_2_ atmosphere at 37°C.

#### 4.1.3. Animals

Male C57BL/6 mice (6 weeks, 20 ± 2 g) were purchased from Tianqin Biotech Co., Ltd. (Changsha, China) and were maintained in an SPF environment. All animal experiment protocols were reviewed and approved by the Experimental Animal Ethics Committee of Central South University and were carried out following the requirements of the National Act on the Use of Experimental Animals (People's Republic of China).

### 4.2. Bioinformatics Analysis

Transcriptome data for the melanoma cohort and immunotherapy cohort were collected from TCGA-SKCM, GEO, and literature searches. The melanoma cohorts includes TCGA SKCM [56] and GEO (GSE133713 [57], GSE98394 [50], GSE54467 [52], GSE22153 [53], and GSE65904 [51]). The immunotherapy cohorts include GSE91061 [55] and Lee et al. [54]. First, we used the MCP method from the IOBR package v0.99.9 to estimate population abundance of tissue-infiltrating immunity [58]. We used univariate COX regression analysis to assess the effect of DC cells score on patient survival, and the Kaplan-Meier survival curve was constructed using survival package v3.3.1. Correlation analysis was conducted to further determine the correlation between DC cells score and T cells score. The Wilcoxon test was used to analyze differences in DC cells score between the benefit and non-benefit groups in the immunotherapy cohort.

### 4.3. Preparation and Characterization of PDM

PST NPs were prepared via self-polymerization of serotonin (5-HT). Briefly, 5-HT·HCl (10 mg, 0.047 mmol) was dissolved in 5 mL ultrapure water and then added to 10 *μ*L NaOH (10 *μ*M) to adjust the pH to 7.0 for 48 h oxidative polymerization. Then, PST NPs were collected via centrifugation (4°C, 16000 rpm, 15 min). To prepare PDM NPs, 200 *μ*L PST NPs (1 mg mL^−1^), 100 *μ*L NaCl (1.5 M), and 20 *μ*L DZ (100 *μ*M) were mixed and incubated for 30 min to adsorb DZ on the surface of PST NPs. Then, 10 *μ*L TA (40 mg mL^−1^) and 10 *μ*L MnCl_2_ (10 mg mL^−1^) were quickly mixed with the above solution. After 5 min stir, 100 *μ*L HEPES (100 mM, pH 7.6) was added and further sonicated for 5 min, and then vigorously stirred at 30°C for 4 h. Finally, the PDM NPs were collected by centrifugation (16000 rpm, 15 min, 4°C) and dispersed in ultrapure water for further use. The ultraviolet-visible (UV-vis) spectra were obtained by using a UV-2600 spectrophotometer (UV-2600, Shimadzu). The size distribution and zeta potential were determined by the Malvern Zeta Sizer Nano series (Nano ZS, Malvern Instruments, UK). The morphology was observed using transmission electron microscopy (TEM) (Titan G2-F20, FEI, USA). The stability in PBS and serum were assessed by measuring particle size over 72 h incubation.

### 4.4. Loading Capacity and Protective Effect of PDM on DZ

To explore DZ loading capacity, different concentrations of FAM-labeled DZ (0.5, 1, 2, and 4 *μ*M) were used to prepare PDM and centrifuged at 20000 rpm for 20 min to collect unloaded DZ, followed by quantification using denaturing PAGE. The gel images were obtained by a gel imaging system (FluorChem Imaging System, Protein-Simple). To measure the protective effect of PDM on DZ, PDM or free DZ (contained 2 *μ*M FAM-labeled DZ) was incubated with 20% FBS (37°C, 3 h), and the samples were heated to denature the FBS (95°C, 15 min). Then, the samples were incubated with 100 mM EDTA to dissociate the TA-Mn^2+^ MOFs and release the DZ. The DZ was analyzed by PAGE electrophoresis as mentioned above.

### 4.5. In Vitro Release of Mn^2+^ and DZ

The in vitro release profile of DZ was analyzed via PAGE electrophoresis. PDM (contained 2 *μ*M FAM-labeled DZ) was dispersed in 2 mL of different media. Then, all samples were placed in a shaker (100 rpm, 37°C). Samples were collected at different time points and centrifuged at 20000 rpm for 20 min. The supernatants were collected for PAGE electrophoresis to analyze DZ. In addition, the Mn^2+^ concentration of supernatants at 48 h was measured by ICP-OES. At 12 h, the precipitation was collected for hydrodynamic diameter measurement and TEM observation.

### 4.6. DZ Activity Assay

The DZ activity of PDM was also accessed via PAGE assay. PDM (contained 2 *μ*M DZ) was added to 1 mL dissolution media (pH 5.5 PBS contained 10 mM GSH) and added 2 *μ*M FAM-labeled substrate strand. Then, the mixture was placed in a shaker (100 rpm, 37°C) and incubated at different time points. Subsequently, 8 M urea was added to quench the reaction, and the supernatants were collected (20000 rpm, 20 min) and further analyzed by 15% PAGE.

### 4.7. Photothermal Effect of PDM

PDM NPs solution (2 mL, 200 *μ*g mL^−1^) was irradiated (808 nm) at different power densities for 600 s. Then, PDM NPs (2 mL) with series concentrations were irradiated (808 nm, 1.5 W cm^−2^) for 600 s. Free PST and MOFs NPs solutions (2 mL, 400 *μ*g mL^−1^, 808 nm, 1.5 W cm^−2^) were also determined for comparison. The temperature was recorded by a digital thermometer at predetermined times. To investigate the photothermal stability of PDM NPs, the PDM NPs solution (2 mL, 400 *μ*g mL^−1^) was irradiated under five laser on/off cycles, and the temperature variation curves were recorded. The photothermal conversion efficiency (*η*) of PDM NPs was calculated as the previous method [59].

### 4.8. PTT-Induced Cytotoxicity and Immunogenic Cell Death In Vitro

In vitro cytotoxicity was evaluated by the MTT assay. B16F10 cells and DC2.4 cells were seeded at a density of 1 × 10^3^ per well into 96-well plates and cultured for 12 h. Then, different formulations were added with or without laser irradiation (808 nm, 1.5 W cm^−2^, 5 min) and incubated for 24 h. Then, the cell viability was measured by MTT protocol. For ICD analysis, B16F10 cells were incubated with PDM NPs (20 *μ*g mL^−1^) with or without irradiation (808 nm, 1.5 W cm^−2^, 5 min). After further 4 h incubation, the media and cells were collected, respectively. Then, proteins were extracted, and Western blot analyses were carried to quantify HSP 70, HMGB1, and GAPDH antibodies. Similar cell experiments were performed to study CRT exposure and ATP release, which was probed by fluorescence inverted microscopy (ECLIPSE-Ti, Nikon) and ATP Assay Kit, respectively.

### 4.9. Antigen Capturing Ability of PDM for Immune Activation

To investigate the antigen capturing ability of PDM, the gradient concentrations of FITC-OVA were added to 200 *μ*g mL^−1^ PST NPs and PDM NPs (pretreated with or without 10 mM pH 5.5 PBS and GSH), respectively, and then co-incubated in a shaker (100 rpm, 37°C) for 4 h. After 10 min centrifugation at 20000 rpm, the fluorescence intensity of OVA (Ex/Em =483 nm/550 nm) in the supernatant of the samples (*m*_*s*_) and the control group (*m*_*c*_) was determined using a microplate reader. The percentage of the adsorbed OVA content (%) was calculated by the equation: (*m*_*c*_ − *m*_*s*_)/*m*_*c*_ × 100%. The zeta potential of NPs before and after incubation with OVA (400 *μ*g mL^−1^) was measured via the DLS instrument.

To evaluate antigen phagocytosis by DCs, DC2.4 were plated at a density of 2 × 10^5^ cells per well in 6-well plates, followed by adding FITC-OVA (free OVA), OVA-captured PST NPs (PST@OVA), and OVA-captured PDM NPs (PDM@OVA). After incubation for 4 h, the cells were fixed in 500 *μ*L 4% paraformaldehyde for 15 min. Subsequently, the cell nuclei were labeled with DAPI for 20 min, and the fluorescent images were obtained using a fluorescence imaging system.

To study the DCs activation and maturation, B16F10 cells (1 × 10^5^ cells mL^−1^) were dispersed in DMEM media and treated with mild heat (43°C) for 5 min to simulate the photothermal-induced cells death and release of antigen. After multi-gelation at -80°C for 4 h, the heat-treated B16F10 cell suspension was centrifuged to obtain the antigen-containing supernatant. The proteins concentration was measured by the BCA Protein Assay kit. Then, the supernatant was incubated with PST NPs or PDM NPs (with or without pretreatment with 10 mM pH 5.5 PBS and GSH) at 37°C for 4 h to obtain antigen-captured NPs. Then, the DC2.4 were incubated with PBS, antigen, or various antigen-captured NPs at a dose of 1 mg mL^−1^ protein for 24 h. Afterwards, the DCs were harvested, and proteins were extracted, followed by Western blot analyses to quantify CD86, MHC II, and GAPDH.

### 4.10. In Vitro *β*-Catenin Silencing and CCL4 Release in B16F10 Cells

B16F10 cells were seeded in 6-well plates at a density of 2 × 10^5^ cells per well for 12 h. The cells were incubated with gradient concentrations of PDM NPs (20, 40, and 100 *μ*g mL^−1^) with or without laser (808 nm, 1.5 W cm^−2^, 5 min) for 24 h. Subsequently, the proteins were extracted, followed by Western blot analyses to quantify *β*-catenin and GAPDH. In addition, the CCL4 levels in the media were detected by the CCL4 ELISA kit.

### 4.11. Preparation and Characterization of PDM MNs

To prepare drug-loaded MNs, PST/MOF or PDM NPs solution (20 mg mL^−1^) was directly deposited onto the PDMS surface by pipetting, followed by vacuum (600 mmHg) for 10 min to flow the solution into the cavities. After that, the redundant solution was removed to make sure each cavity was filled with drug solution and then evaporated for 10 min to concentrate drugs on MN tips. Subsequently, 40% (wt%) PVP K30 and 15% (wt%) HA matrixes were deposited onto each micro mold surface to form the base. The final formulation was dried in a sealed desiccator overnight and stored in a desiccator. The morphology of MNs was characterized by the scanning electron microscope, and drug loading was calculated by dissolving MNs in a certain volume of PBS.

The mechanical property of MNs was assessed using a universal testing machine. To estimate the strength of MNs, the isolated abdominal skins of C57BL/6 mice were fixed on a board, and then the drug-loaded MN patch was inserted into the skins. After insertion, the patch was removed, and a digital camera was used to observe the skins and calculate the insertion ratio. Then, the skins were excised to determine the insertion depth by frozen tissue sections. Finally, the MNs were inserted into the skin using a thumb for 2 min. After removing the MNs, the skin surface was monitored at different time points to access the recovery of the skins.

The skin penetration of the MNs was evaluated by using Franz transdermal diffusion cell. Briefly, the MNs were applied to depilated back skin via thumb insertion for 5 min, and then the skin was transferred to Franz transdermal diffusion cell (the transdermal area was 2.50 ± 0.08 cm^2^) with receiving cell containing 2.1 mL phosphate-buffered saline solution (pH 6.8, 200 rpm, 37°C). At different time point, the receiving solution was collected, and EDTA (100 mM) was added to dissociated PDM. After centrifugation, the FAM-labeled DZ in solution was measured to calculate the penetration of PDM.

The recovery of the skin after MNs treatment was studied on the back skin of C57 mice. The MNs were inserted into the skin using the thumb and index finger for 5 min. After removing the MNs, the morphological changes of the skin were monitored at different time points. Moreover, after laser irradiation at 24 h post-administration, the mice were sacrificed and the skins were collected for H&E staining.

### 4.12. Photothermal Effect of the MNs

The MNs were placed with the needle tips facing up. The distance between the MNs and the spotlight was fixed at 10 cm, and the spot size was ~1 cm^2^. Surface temperature changes of the MN patch backing were recorded (FLIR E4). To evaluate the photothermal effect of MNs in vivo, the hair of mice at the dorsal flank was removed, and MNs were inserted into the skin by thumb pressure for 5 min. At 24 h post-injection, the laser was performed, and the photothermal effect was evaluated.

### 4.13. Antitumor Effect In Vivo

For the antitumor effect study, 1 × 10^6^ B16F10 cells were subcutaneously injected into 6-week-old male C57BL/6 mice to establish the orthotopic melanoma mice model. For the anti-abscopal effect study, the mice were subcutaneously injected with 1 × 10^6^ B16F10 cells in the right posterior dorsal as the first tumor, and then 1 × 10^6^ B16F10 cells were seeded in the left posterior dorsal at 6 days later as the second tumor. When the volume reached 50~100 mm^3^ (after around 7 days), mice were randomly divided into five groups (*n* =6) and applied with various treatments (every three days for 3 times) on the skin of tumor site (for the abscopal tumor, treatment was performed only for left-side tumor): blank, blank MN, PST/MOF MNs + L (containing PST/MOF NPs), PDM MNs, and PDM MNs + L (+ L represented the irradiation with a laser). The groups with NIR laser irradiation (808 nm, 1.5 W cm^−2^) were performed at 24 h post-administration for 5 min. The tumor size and body weight were recorded every three day, and the tumor volume (V) was calculated according to the formula: V = length × width^2^/2. On day 12, mice were sacrificed, and tumor and major organs were collected for photographing, weighing, and immunohistochemistry analysis. In addition, the serum was collected for analysis. The tumor slices were collected for CRT expression, *β*-catenin expression, and CCL4 analysis. The tumors were also examined for the levels of CD8^+^ T cells and granzyme positive CTLs by flow cytometry and immunohistochemical analysis. The levels of DCs and mature DCs in tumor and lymph nodes were analyzed via flow cytometry and immunohistochemical analysis. The serum and tumor tissue samples were collected to examine IL-6, IFN-*γ*, and IL-12 using ELISA kits.

For immunohistochemical assay, tumors were stained according to the standard protocol. The images of immunohistochemical results were analyzed by Image J 1.53c software. For immunofluorescence assay, the tissue sections were handled according to the immunohistochemical procedure until the primary antibody was incubated. Slides were incubated with HRP second antibody for 30 min and then incubated with TYR-488 for 15 min. The sections were again subjected to the following procedures: antigen repair, blocking, incubation with H_2_O_2_, and another primary antibody incubation for one night. Then, the sections were incubated with HRP second antibody for 30 min, incubated with TYR-CY3, and incubated with DAPI solution at room temperature for 10 min. In this procedure, the slices were cleaned with PBS-0.1% Tween 20 three times between each step. The section was cover slipped with an anti-fade mounting medium. Images were detected and captured by Fluorescent Microscopy (Nikon, ECLIPSE Ts2R). The fluorescent intensity was analyzed with Image J 1.53c software.

For flow cytometry analysis, the single-cell suspension of mice tumor was obtained by rapid and gentle stripping, physical grinding, and filter filtration. After blocking with CD16/CD32 antibody, cells were stained with Zombie Red Fixable Viability Kit (77475), followed by staining with GZMB, CD4, CD8, CD3, and CD45 antibodies for 20 min to analyze T cell, or staining with I-A/I-E, CD40, CD11C, and CD45 antibodies to analyze DC cell. After fixation and permeabilization with True-Nuclear Transcription factor Buffer Set, the cells were stained with GZMB antibody for intracellular protein. Stained cells were analyzed by FACS Dxp AthenaTM (Cytek, Fremont, CA, USA). Data were further analyzed by Flow Jo 10.0 software.

For flow cytometry analysis, cells in tumors were selected using the following gating strategy. FSC and SSC were used to select main population. SSC and zombie box were used to select live cells. Then, SSC and CD45 were used to select CD45^+^ immune cell populations. SSC and CD3 were used to select CD3^+^ T cells. Finally, CD8 or CD8/GZMB was used to analyze CD8^+^ or CD8^+^GZMB^+^ T cell populations (Figure S20A). Cell populations in lymphonodus were also selected by using FSC/SSC and zombie/SSC. SSC and CD45 were used to select CD45^+^ immune cell population. MHC-II(I-A/I-E) and CD11c were used to select DC cells. CD40 or CD103 was used to analyze CD40^+^ or CD103^+^ DC cell population.

### 4.14. Statistical Analysis

Statistical analyses were performed using GraphPad Prism 6.01 software. One-way analysis of variance (ANOVA) was used to assess the significance of differences among groups. The significance was defined as follows: ∗*P* < 0.05, ∗∗*P* < 0.01, ∗∗∗*P* < 0.001, and ∗∗∗∗*P* < 0.0001.

## Data Availability

All data that support the findings of this study are available from the corresponding author upon reasonable request.

## Supplementary Materials

Supplementary Materials Figure S1: KM survival curve in melanoma cohort. Figure S2: Stability of the NPs. Figure S3: EDS analysis of NPs. Figure S4: Release profiles of the NPs. Figure S5: Characterizations of the NPs after treatment with PBS (pH 5.5) buffer containing GSH. Figure S6: Cleavage activity of DZ. Figure S7: Photothermal characterization of the NPs. Figure S7: Characterizations of NPs for OVA adsorption. Figure S9: Effect of temperature on DZ activity. Figure S10: Transdermal penetration of the NPs. Figure S11: H&E staining of skins after various treatments. Figure S12: Immunohistochemical staining and quantification of CRT in tumor tissue. Figure S13: Immunohistochemical staining and quantification of *β*-catenin in tumor tissue. Figure S14: Analysis the matured CD103+ DCs (CD103+CD11c+MHCII+) in lymph nodes. Figure S15: Analysis the DCs (CD40+CD11c+MHCII+) in lymph nodes for primary tumor. Figure S16: Analysis the cytotoxic T lymphocytes (CTLs, CD3+CD8+) in tumors. Figure S17: Analysis the cytotoxic T lymphocytes (CTLs, CD3+CD8+) in tumors. Figure S18: Analysis the DCs (CD40+CD11c+MHCII+) in lymph nodes for the 2nd tumors. Figure S19: Activation of T cells to characterize the abscopal antitumor effect. Figure S20: The gating strategy of the flow cytometry.
