# Supplementary material for: Photothermal Nano-Vaccine Promoting Antigen Presentation and Dendritic Cells Infiltration for Enhanced Immunotherapy of Melanoma via Transdermal Microneedles Delivery
Source: Research (Wash D C). 2022 Sep 2;2022:9816272. doi: 10.34133/2022/9816272 (PMC9484834; doi:10.34133/2022/9816272)
Supplement: Supplementary Materials — Figure S1: KM survival curve in melanoma cohort. Figure S2: Stability of the NPs. Figure S3: EDS analysis of NPs. Figure S4: Release profiles of the NPs. Figure S5: Characterizations of the NPs after treatment with PBS (pH 5.5) buffer containing GSH. Figure S6: Cleavage activity of DZ. Figure S7: Photothermal characterization of the NPs. Figure S7: Characterizations of NPs for OVA adsorption. Figure S9: Effect of temperature on DZ activity. Figure S10: Transdermal penetration of the NPs. Figure S11: H&E staining of skins after various treatments. Figure S12: Immunohistochemical staining and quantification of CRT in tumor tissue. Figure S13: Immunohistochemical staining and quantification of β-catenin in tumor tissue. Figure S14: Analysis the matured CD103+ DCs (CD103+CD11c+MHCII+) in lymph nodes. Figure S15: Analysis the DCs (CD40+CD11c+MHCII+) in lymph nodes for primary tumor. Figure S16: Analysis the cytotoxic T lymphocytes (CTLs, CD3+CD8+) in tumors. Figure S17: Analysis the cytotoxic T lymphocytes (CTLs, CD3+CD8+) in tumors. Figure S18: Analysis the DCs (CD40+CD11c+MHCII+) in lymph nodes for the 2nd tumors. Figure S19: Activation of T cells to characterize the abscopal antitumor effect. Figure S20: The gating strategy of the flow cytometry. [file 9816272.f1.docx]

**Supporting Information**

**Integrated Microneedles patches as Photothermal-Derived Antigen trapper for Photoimmunotherapy of Melanoma**

Jiaojiao Zhu^1+^, Ruimin Chang^2, 3, 4+^, Benliang Wei^2, 3^, Yao Fu^2, 3, 4^, Xiang Chen*^2, 3^, Hong Liu*^2, 3^, Wenhu Zhou*^1^

^1.^ Xiangya School of Pharmaceutical Sciences, Central South University, Changsha, Hunan, 410013, China

^2.^ Department of Dermatology, Hunan Engineering Research Center of Skin Health and Disease, Hunan Key Laboratory of Skin Cancer and Psoriasis, Xiangya Hospital, Central South University, Changsha 410008, Hunan, China

^3.^ National Engineering Research Center of Personalized Diagnostic and Therapeutic Technology, Xiangya Hospital, Central South University, Changsha 410008, Hunan, China

^4.^ Department of Thoracic Surgery, Xiangya Hospital, Central South University, Changsha, Hunan, 410008, China.

[^+^] These authors contributed equally to this work.

*Email: [zhouwenhuyaoji@163.com](mailto:zhouwenhuyaoji@163.com); [chenxiangck@126.com](mailto:chenxiangck@126.com); [Hongliu1014@csu.edu.cn](mailto:Hongliu1014@csu.edu.cn)


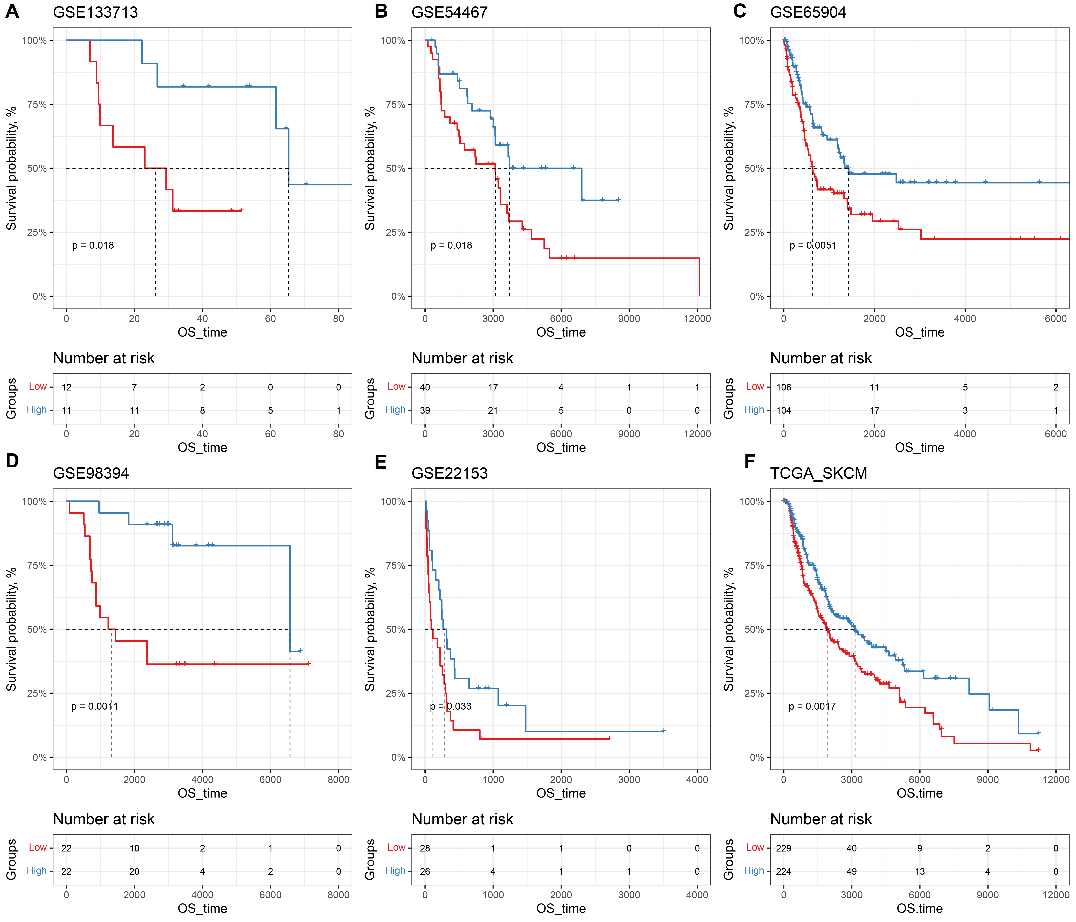


**Figure S1.** A-F) KM survival curve in melanoma cohort.


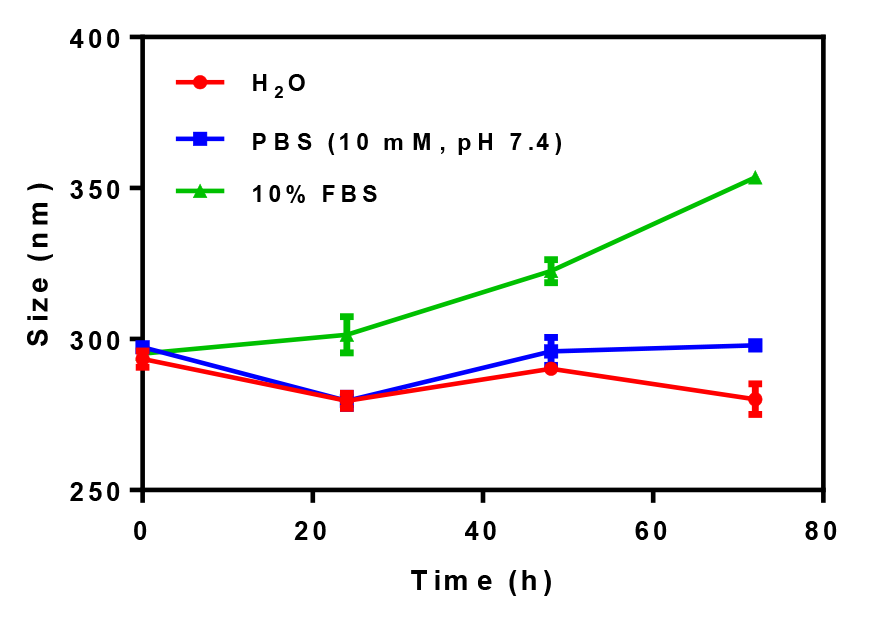


**Figure S2.** The stability of PDM NPs in different conditions.


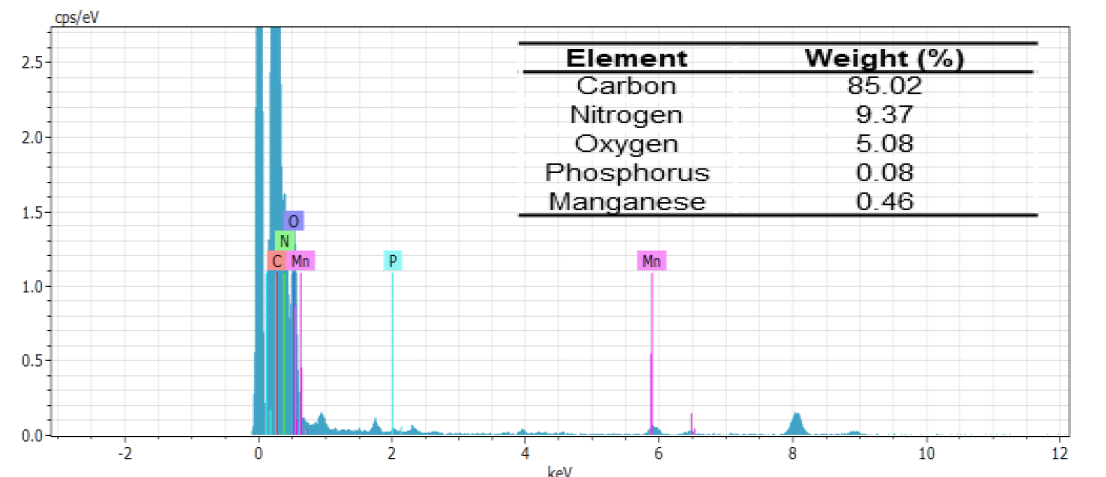


**Figure S3.** energy disperse X-ray spectroscopy (EDS) analysis of PDM NPs.


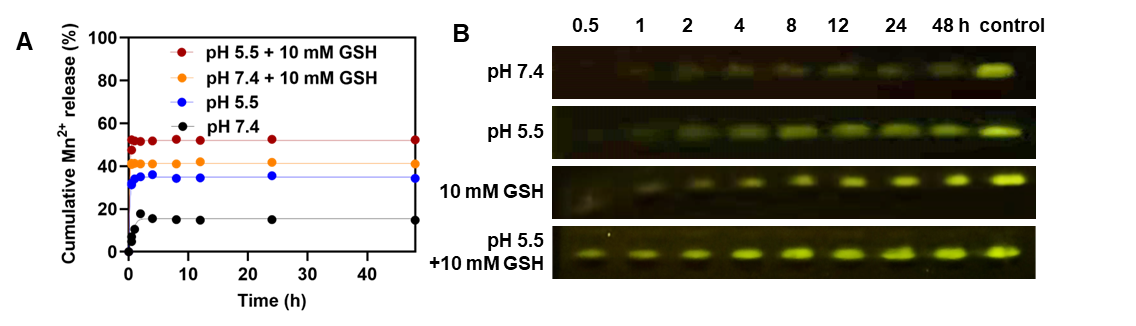


**Figure S4.** A) Release of Mn^2+^ from PDM under various conditions by ICP MS. B) PAGE gel images for characterization of DZ release from PDM under various conditions.


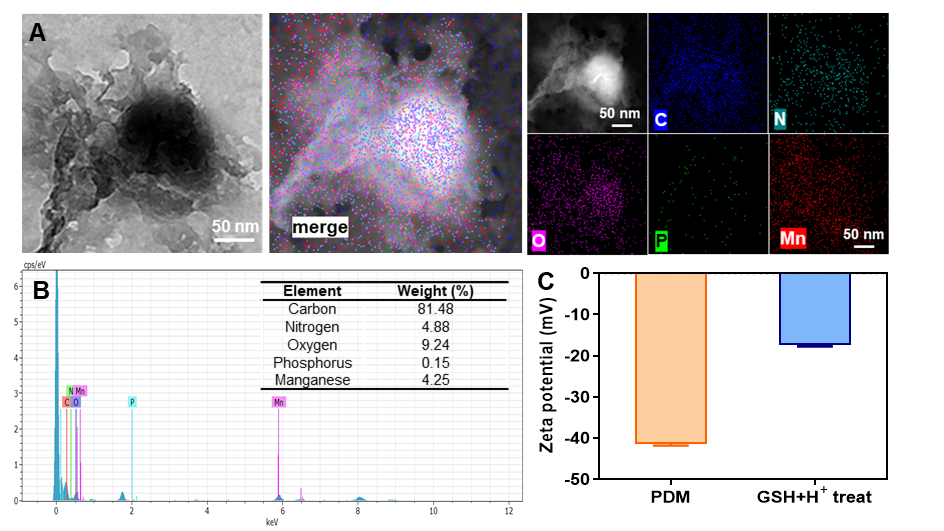


**Figure S5.** A) TEM image, elemental mapping image, B) energy disperse X-ray spectroscopy (EDS) analysis and C) the zeta potential of PDM NPs after pH 5.5 PBS buffer containing GSH treatment.

**
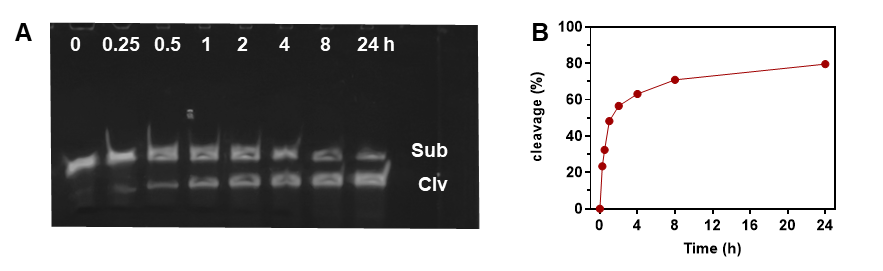
**

**Figure S6.** A) PAGE image for characterization of DZ activity for substrate cleavage. B) The cleavage quantification.


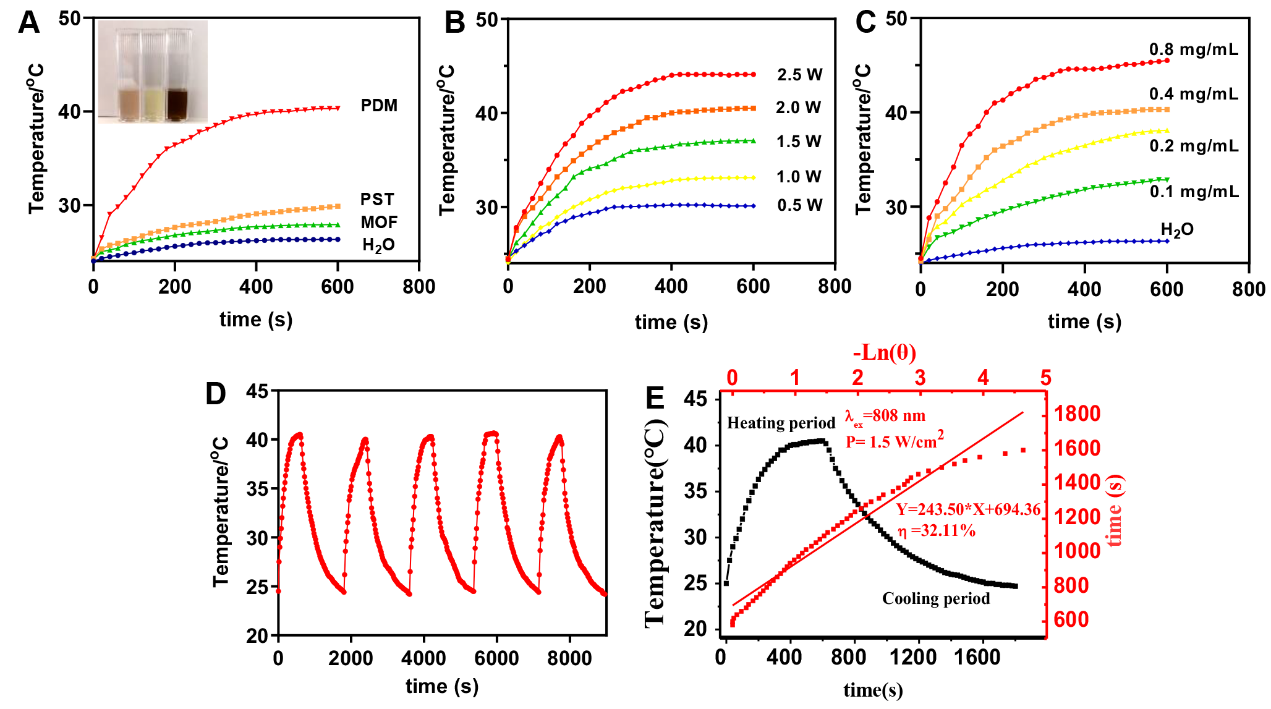


**Figure S7.** A) Heating curves of PHT, MOF, and PDM at 400 μg mL^-1^ irradiated with fixed laser power (808 nm, 1.5 W cm^−2^). Inset: corresponding photographs of PHT, MOF, and PDM solution. B) Heating curves of PDM at 200 μg mL^-1^ irradiated with different power density. C) Heating curves of PDM at different concentrations irradiated with fixed laser power (808 nm, 1.5 W cm^−2^). D) Photothermal stability curves of PDM at 400 μg mL^-1^ irradiated by NIR laser at a power density of 1.5 W cm^−2^ for five cycles. E) Calculation of the photothermal conversion efficiency at 808 nm. Red line: time constant (τ s) for the heat transfer from the system determined by applying the linear time data from the cooling period.


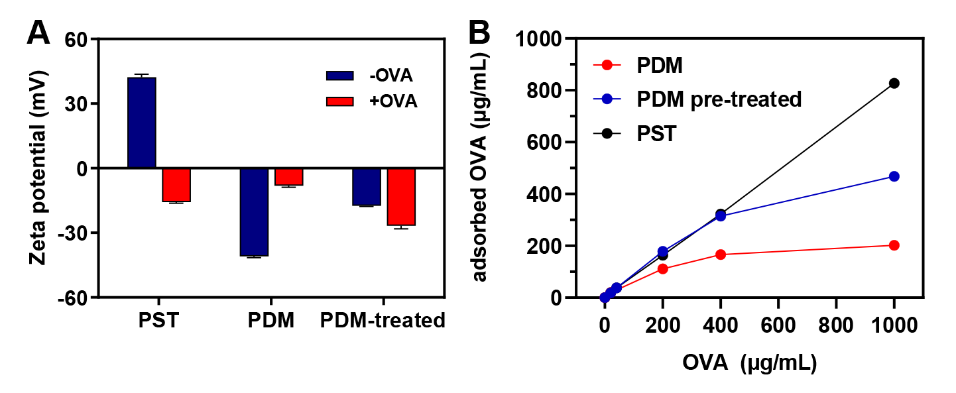


**Figure S8.** A) The size distribution and B) the zeta potential of PHT, PDM and PDM-treated with or without OVA adsorption. C) The OVA adsorption capacity of different NPs.


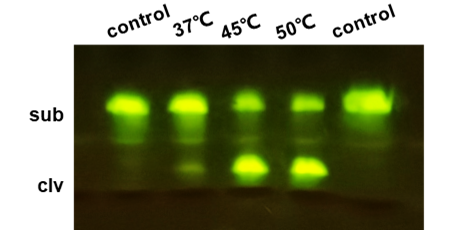


**Figure S9.** PAGE gel image to analyze the effect of temperature on DNAzyme activity. The “sub” and “clv” indicated the band of full-length substrate and the cleaved product, respectively.


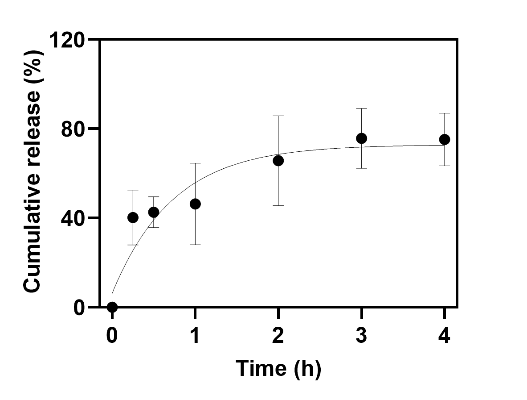


**Figure S10.** Dynamic monitoring the transdermal penetration of PDM MNs by Franz diffusion cell. For nanoparticles quantification, the loaded DZ was labeled with a FAM fluorophore.


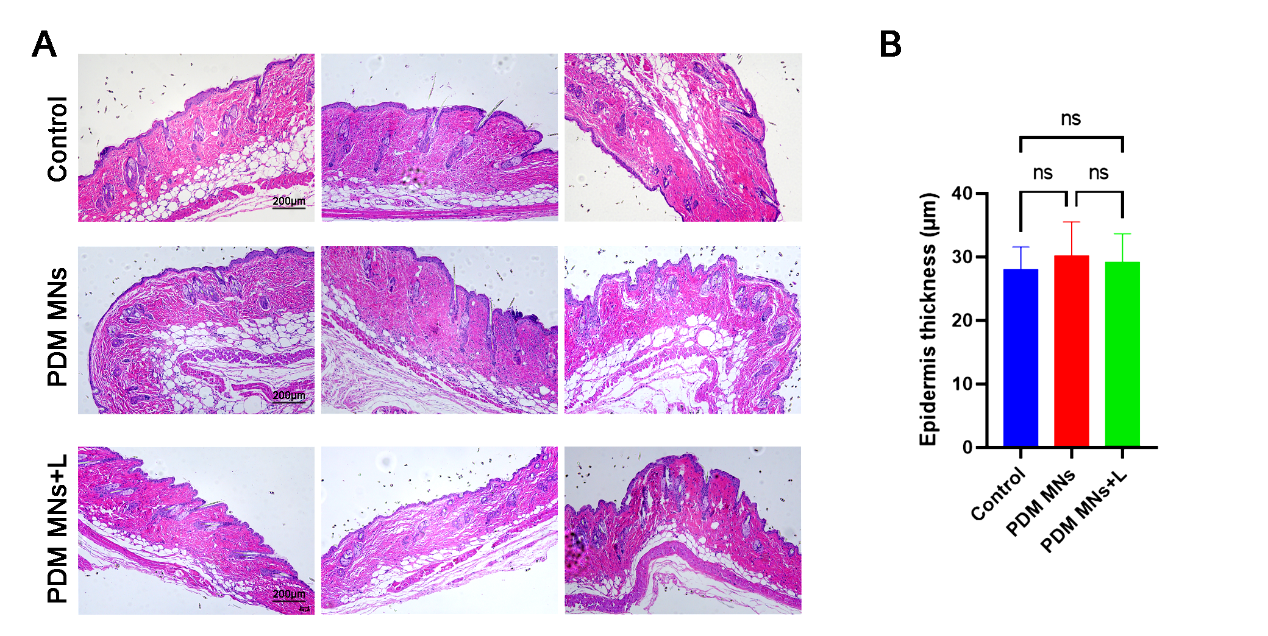


**Figure S11.** H&E staining of skins after various treatments. A) H&E staining of skin section of mouse treated with PDM MNs or PDM MNs + L or control mouse. (B) Epidermis thickness measured from sections of H&E staining.


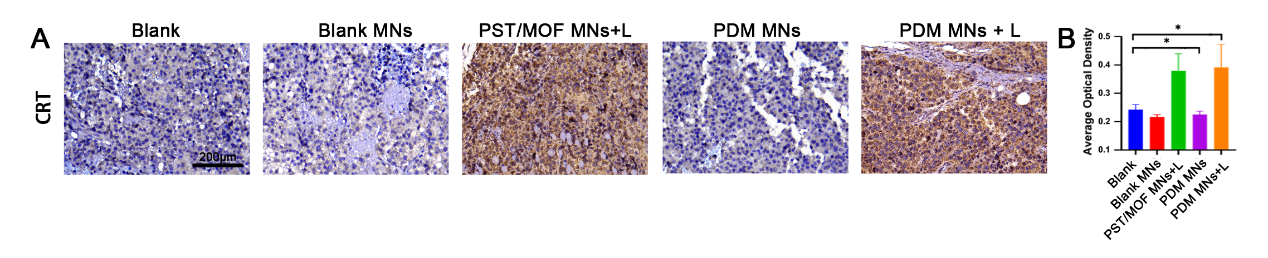


**Figure S12.** A) Immunohistochemical staining of CRT in tumor tissue and (B) the relative quantified results. Scale bar: 100 μm.


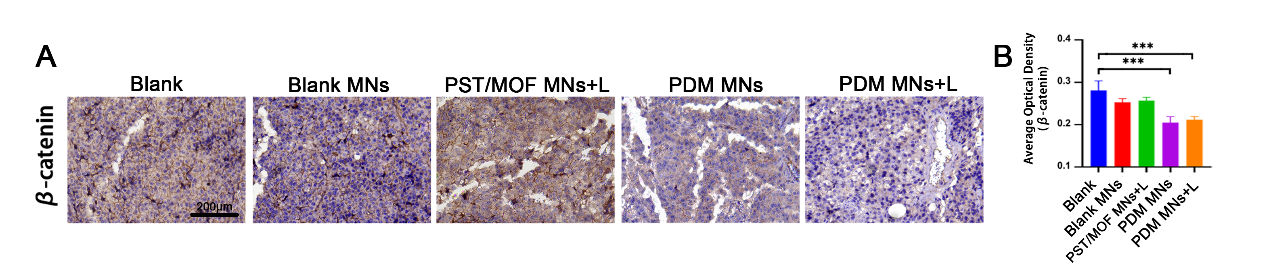


**Figure S13.** A) Immunohistochemical staining of β-catenin in tumor tissue and (B) the relative quantified results. Scale bar: 100 μm.


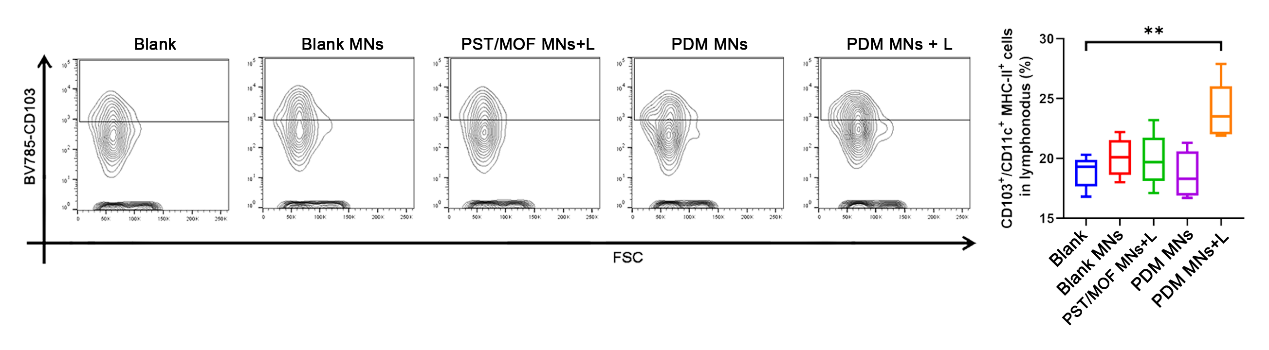


**Figure S14.** A) Representative flow cytometry images and B) the percentage of the matured CD103^+^ DCs (CD103^+^CD11c^+^MHCII^+^) in lymph nodes after various treatments.


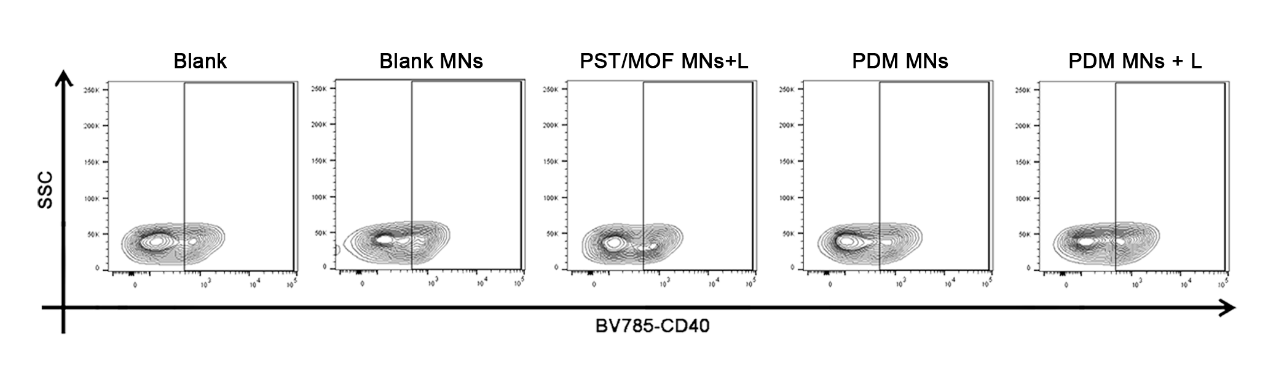


**Figure S15.** A) Representative flow cytometry images and B) the percentage of the matured DCs (CD40^+^CD11c^+^MHCII^+^) in lymph nodes after various treatments.


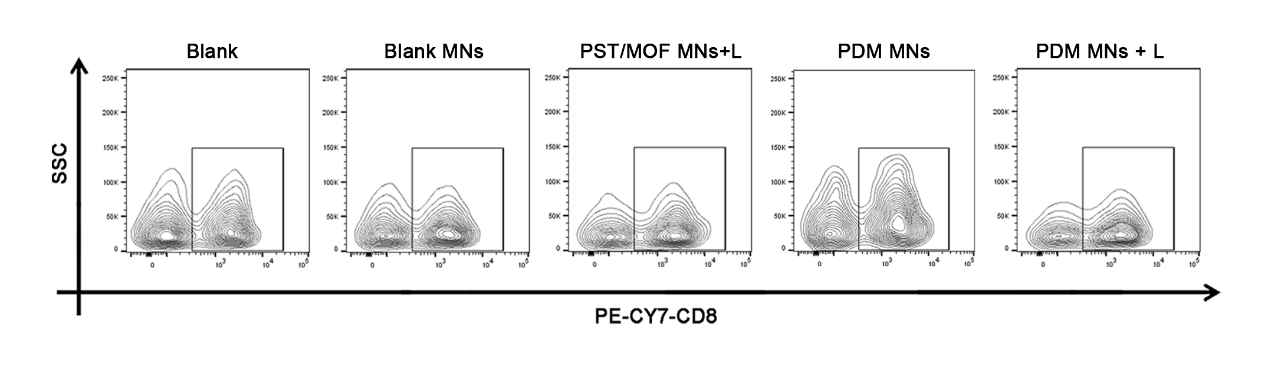


**Figure S16.** A) Representative flow cytometry images and B) the percentage of the cytotoxic T lymphocytes (CTLs, CD3^+^CD8^+^) in tumors after various treatments.


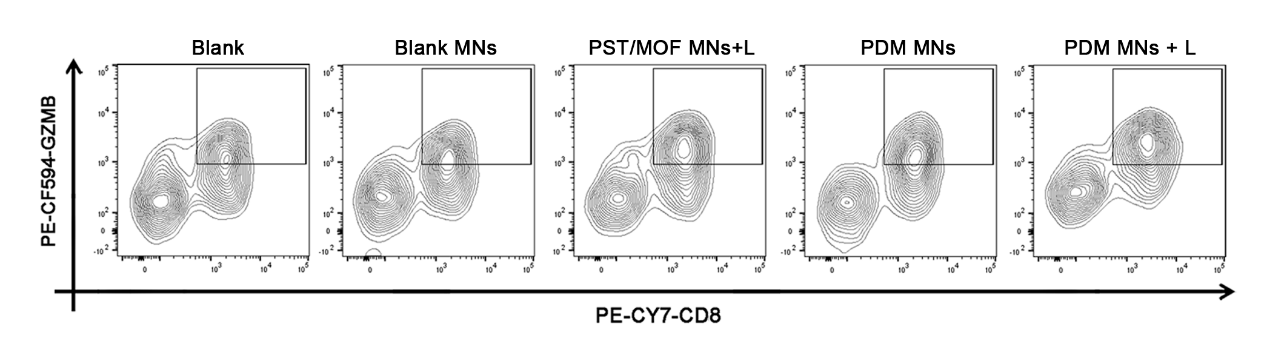


**Figure S17.** A) Representative flow cytometry images and B) the percentage of granzyme B positive CTLs (CD3^+^CD8^+^GZMB^+^) in tumors after various treatments.


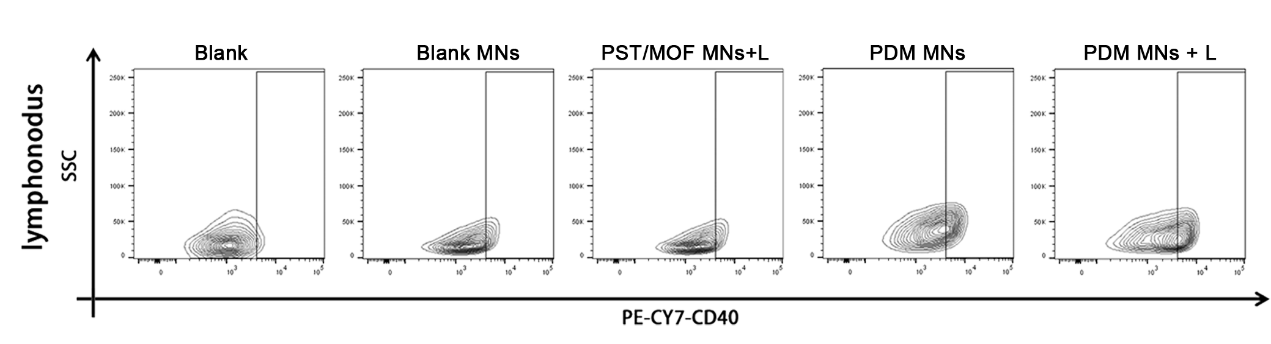


**Figure S18.** A) Representative flow cytometry images and B) the percentage of the matured DCs (CD40^+^CD11c^+^MHCII^+^) in lymph nodes after various treatments.


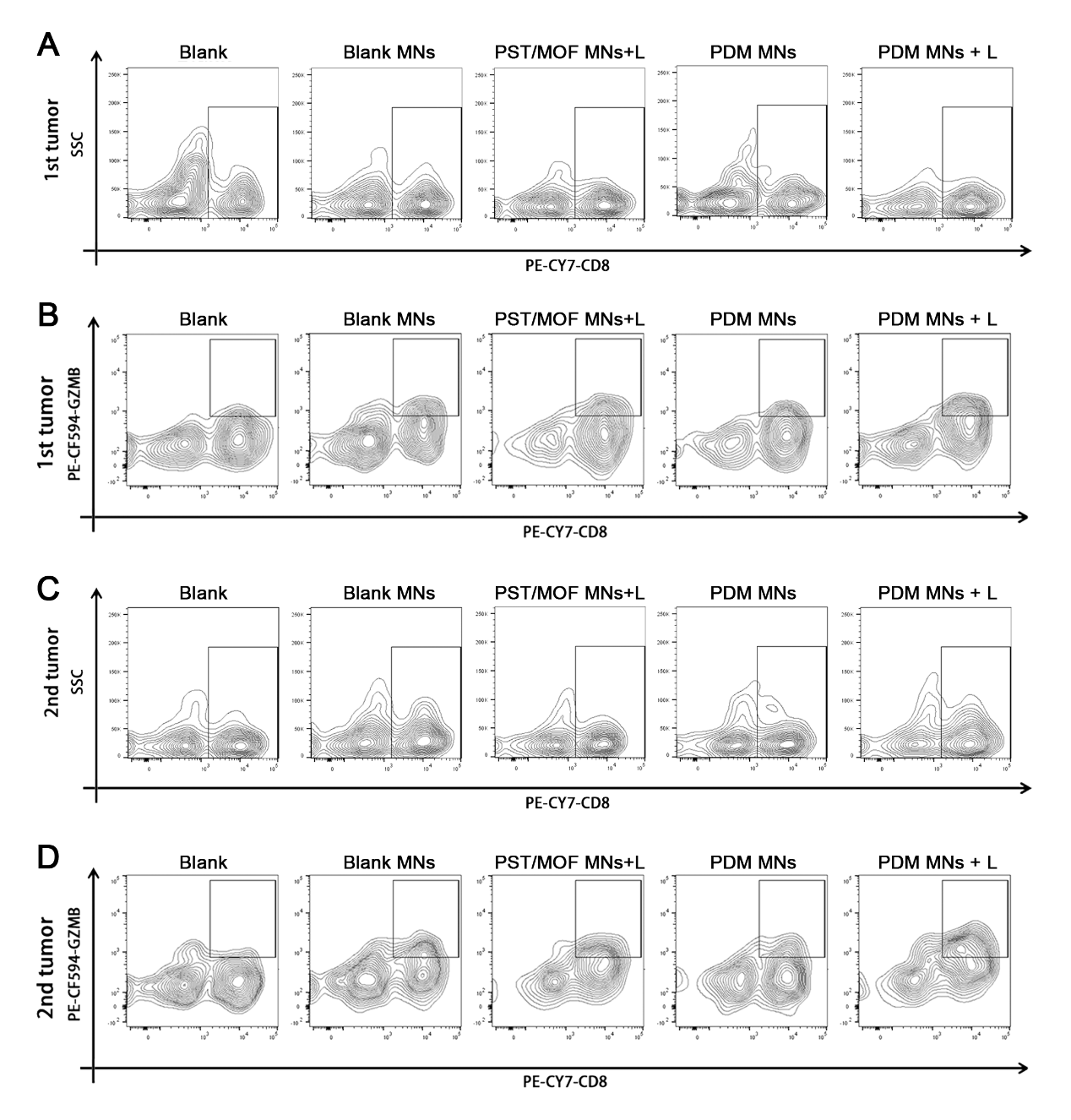


**Figure S19.** Representative flow cytometry images of A) cytotoxic T lymphocytes (CTLs, CD3^+^CD8^+^) and B) granzyme B positive CTLs (CD3^+^CD8^+^GZMB^+^) in 1^st^ tumors after various treatments. Representative flow cytometry images of C) cytotoxic T lymphocytes (CTLs, CD3^+^CD8^+^) and D) granzyme B positive CTLs (CD3^+^CD8^+^GZMB^+^) in 2^nd^ tumors after various treatments.


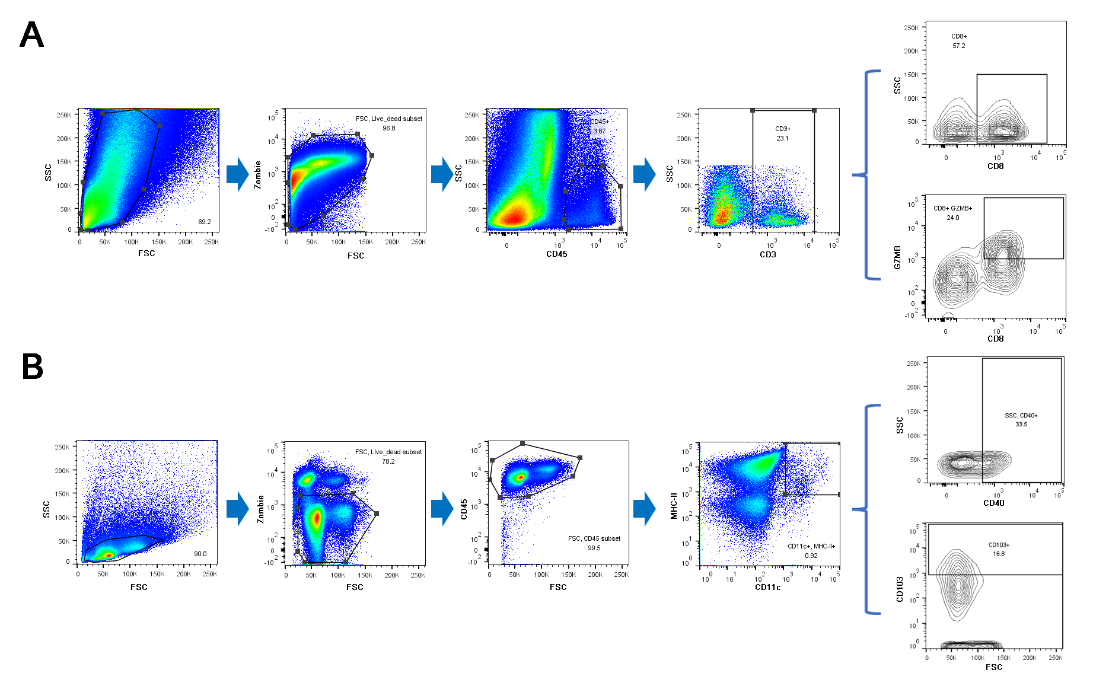


**Figure S20.** The gating strategy of the flow cytometry. A. The gating strategy for CD8+ T cells and CD8+/GZMB+ T cells in the tumor. B. The gating strategy for CD40+ and CD103+ DC cells in the lymphonodus.
